# Biotic Stress Resistance in Sweet Potato: Mechanisms, Perspectives, and Sustainable Production Strategies

**DOI:** 10.3390/plants15101504

**Published:** 2026-05-15

**Authors:** Hai Zheng, Jiachun Weng, Liehong Wu, Zhixian Ji, Yusha Meng, Shengfa Shen, Chao Xiang

**Affiliations:** 1 Institute of Crops and Nuclear Technology Utilization, Zhejiang Academy of Agricultural Sciences, Hangzhou 310021, China; 2 College of Food and Health, Zhejiang Agriculture and Forestry University, Hangzhou 311300, China

**Keywords:** sweet potato, biotic stress, sustainable production, resistance mechanisms, breeding strategies

## Abstract

Food security is increasingly threatened by climate change and population growth. Sweet potato has become a crucial crop for ensuring food security due to its adaptability to marginal lands and high yield potential. However, its sustainable production is severely limited by diverse biotic stresses (including fungi, viruses, nematodes, insect pests and bacteria), which cause substantial yield losses. Despite its considerable importance, the key bottlenecks in this field remain unresolved, including the incomplete elucidation of core resistance mechanisms, unclear molecular regulatory networks underlying defense responses, insufficient understanding of crosstalk among multiple stresses, and limited integration of emerging technologies into practical resistance breeding. This review synthesizes the latest advances over the past two years. We dissect sweet potato’s defense mechanisms from multiple dimensions and provide novel insights into biotic stress resistance gene regulatory networks. Given that sweet potato production faces the combined effects of multiple pests and biotic-abiotic stresses, we elaborate on the complex stress interactions in sweet potato. In addition, we propose biotic stress management strategies and a ten-year cultivar improvement roadmap that leverages the potential of emerging technologies, including artificial intelligence (AI), gene editing, novel omics approaches and synthetic biology. Taken together, with continuous intensification of global biotic stress challenges, systematic multi-dimensional strategies are imperative to alleviate biotic stress-associated yield and quality impairment in sweet potato. On this basis, this review provides a valuable theoretical and practical reference for resistance breeding and the sustainable production of sweet potato.

## 1. Introduction

With global climate change and growing population pressure, ensuring food security has become a critical issue for sustainable development [1,2,3]. Increasingly frequent extreme climate events, such as high temperature and drought, are progressively reducing the areas suitable for cultivation of major staple food crops [4,5,6,7,8]. Consequently, the utilization of marginal lands (e.g., saline-alkali soils, drought-prone lands and barren lands) has become an important strategy to alleviate arable land shortages. In this context, stress-tolerant crops with broad environmental adaptability are increasingly important [9,10]. Globally, sweet potato is an important crop that possesses remarkable agronomic advantages, including drought tolerance, adaptability to marginal lands, a short growth cycle and stable high yield potential [11,12]. These advantages enable sweet potato to thrive on marginal lands where staple crops grow poorly, making it an essential crop for ensuring food security. Thus, sweet potato plays an important role in global food security and poverty reduction [13,14,15,16]. Beyond serving as a staple food, sweet potato also holds significant economic value. It serves not only as livestock feed but also as a source of raw materials for industrial products such as starch and ethanol [17,18,19].

Despite the importance of sweet potato, its sustainable production is challenged by various biotic stresses, such as fungi, bacteria, viruses, nematodes and insect pests. These biotic stresses often cause severe damage to sweet potato growth, storage root development and postharvest quality, resulting in substantial yield losses and increased costs worldwide [20,21,22,23,24,25,26,27]. In recent years, the frequency and severity of these biotic stresses have been further exacerbated by factors such as climate change, germplasm exchange, and cropping practices. Consequently, the severe impact of biotic stresses on sweet potato production has attracted considerable research attention.

**Figure 1:**
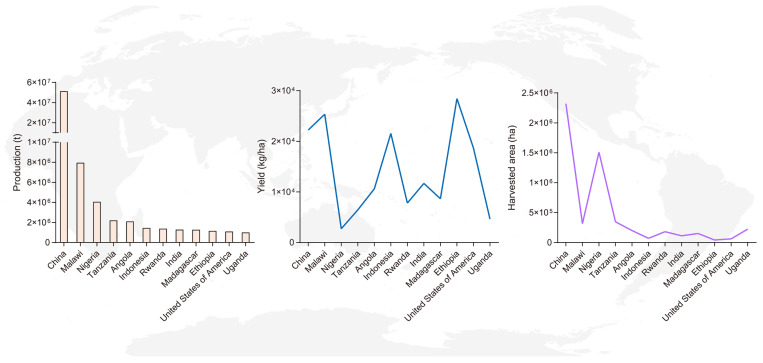
Sweet potato production, yield and harvested area in major sweet potato-producing countries (production exceeding one million metric tons) that reported data for 2024. Pale yellow boxes represent production quantities (t, t = ton), dark blue lines indicate yield (kg/ha, kg = kilogram, ha = hectare) and purple lines indicate harvested area (ha). Data source: Food and Agriculture Organization of the United Nations.

According to data released by the Food and Agriculture Organization of the United Nations (FAO) for 2024, the global harvested area of sweet potato surpassed 7 million hectares, with total production exceeding 90 million metric tons. However, sweet potato yields exhibit significant regional variations (Figure 1). This gap poses a serious threat to global food security and sustainable development. The factors underlying these disparities are multifaceted. In addition to challenges such as relatively outdated cultivation techniques, inadequate genetic improvement of cultivars and suboptimal soil quality in developing countries, severe biotic stresses are considered one of the key constraints limiting yield improvement [28,29]. For example, in Africa, farmers’ insufficient perception and practices hinder the effective management of sweet potato virus disease (SPVD) [30]. Furthermore, while pests and diseases are generally well controlled in developed countries using approaches such as clean planting materials and pheromone traps to inform insecticide application, applying these methods in tropical regions (mostly developing countries) is often impractical [31,32]. Additionally, the high temperature and high humidity conditions prevalent in tropical regions accelerate the spread of pathogens and insect pests and intensify the damage they cause, thereby limiting the sustainable production of sweet potato [33,34,35,36]. Overall, despite its strong environmental adaptability, sweet potato production remains vulnerable to diverse biotic stresses. Consequently, elucidating the mechanisms underlying biotic stress resistance in sweet potato and developing sustainable management strategies are essential, especially in developing regions.

This review summarizes the major biotic stresses affecting sweet potato production and synthesizes the current state of knowledge on the mechanisms underlying its biotic stress resistance. We discuss the impact of climate change on biotic stress severity and the spread pathways of pathogens and insect pests. Additionally, we highlight the critical biotic stress challenges facing sustainable production of sweet potato. To address these challenges, we further propose a series of key research directions and coping strategies for future studies. These efforts will facilitate the cloning of resistance-related genes, clarify the regulatory molecular networks, optimize cultivation practices and enhance biotic stress resistance breeding in sweet potato, thereby contributing to its sustainable production.

## 2. Major Biotic Stresses in Sweet Potato: Types, Distribution and Spread

As elaborated earlier, despite the remarkable adaptability of sweet potato, its global production is threatened by diverse biotic stresses, including fungi, viruses, nematodes, insect pests and bacteria, which result in substantial yield losses and quality deterioration annually, posing a serious challenge to the sustainability of sweet potato production and global food security (Table A1).

### 2.1. Types of Biotic Stresses

Fungal diseases are one of the major constraints to sweet potato production. *Fusarium* root rot, *Fusarium* wilt and black rot are globally prevalent in sweet potato cultivation areas, inducing storage root rot and plant wilting, which results in significant yield losses during disease outbreaks [37,38,39,40,41,42]. Thus, these diseases pose a persistent threat to sweet potato from seedling establishment to postharvest storage. Fungal pathogens are capable of long-term persistence in soil and can be disseminated through diverse pathways such as soil, irrigation water, agricultural machinery and commercial trade, which greatly complicates disease management [42,43,44]. *Fusarium* root rot, a devastating soil-borne disease, has spread rapidly and become a major constraint on sweet potato production, with a high incidence in fields under long-term continuous cropping. This disease is primarily caused by soil-borne fungal pathogens, with *Fusarium solani* as one of the dominant causal agents. These pathogens infect the sweet potato root systems, resulting in necrosis and rot of storage root tissues, thereby impeding the uptake of water and nutrients [42,45,46,47]. *Fusarium* wilt, another major soil-borne disease, is primarily caused by *Fusarium oxysporum*. This pathogen colonizes the vascular tissues of sweet potato, blocks water and nutrient translocation, and ultimately causes leaf wilting and plant death [29,48]. In contrast to *Fusarium* root rot and *Fusarium* wilt, black rot, caused by *Ceratocystis fimbriata*, is a highly destructive postharvest disease [40]. The disease is characterized by the formation of black, firm and sunken lesions on the storage root surface, which enlarge as the disease progresses. Notably, even slightly infected storage roots are prone to severe decay during storage, resulting in substantial economic losses [40,49].

Viral infections, characterized by symptoms such as leaf curling and stunted growth, not only impair plant growth but also spread readily through storage roots, thereby posing another major challenge to sustainable sweet potato production [50,51,52,53,54,55,56]. More than 30 types of DNA and RNA viruses have been identified as capable of infecting sweet potato [57]. Sweet potato feathery mottle virus (SPFMV) is one of the most widespread viruses affecting sweet potato production globally [58,59]. Additionally, sweet potato chlorotic stunt virus (SPCSV) and sweet potato virus G (SPVG) are also significant viruses affecting sweet potato production [60,61,62]. Notably, compared to single-virus infection, synergistic viral infection leads to more severe disease symptoms. The most typical example is the co-infection of SPCSV and SPFMV, which leads to SPVD, the severe viral disease of sweet potato [53,63]. However, despite the serious threat of SPVD to sweet potato production, an effective method for genetic improvement for SPVD resistance remains lacking to date [64].

A broad spectrum of nematode species inflicts damage on sweet potato, such as stem nematode (*Ditylenchus destructor* Thorne), southern root-knot nematode (*Meloidogyne incognita*) and reniform nematode (*Rotylenchulus reniformis*). Stem nematode is recognized worldwide for its extensive distribution and destructive impact, and is particularly prevalent in temperate regions of China, including Hebei, Shandong and Henan provinces. Stem nematode inflicts substantial damage on the storage roots. Sweet potato yield losses attributable to this nematode typically range from 20% to 50%, with complete crop failure occurring under severe disease pressure [65,66,67]. In addition, stem nematode exhibits robust environmental adaptability, enabling it to survive under low-temperature conditions. These characteristics considerably complicate the management of stem nematode. Stem nematode enters sweet potato tissues through stomata or wounds and releases enzymes that soften cell walls to feed on stem and root cells [68]. This process leads to the formation of lesions and cavities in the root, thereby impairing plant growth and development. Similar to stem nematode, southern root-knot nematode is a plant-parasitic nematode that is difficult to control, owing to its short life cycle, wide host range and few effective control measures [69]. In sweet potato, symptoms caused by southern root-knot nematode resemble those of abiotic stresses, such as stunted growth, wilting, leaf discoloration and root malformation. This creates difficulties in accurately identifying this pest. Reniform nematode is highly aggressive on sweet potato, with reports indicating that a population density of five nematodes per 100 cm^3^ of soil can adversely affect both the quality and yield of sweet potato [70]. The management of this nematode in sweet potato cultivation presents significant challenges due to the lack of host resistance and the environmental and health concerns associated with fumigants.

Bacterial pathogens can induce vascular damage, leaf yellowing and abscission, impairing nutrient translocation and photosynthetic efficiency [26,71,72,73]. Although bacterial diseases are less common than fungal diseases, nematode diseases and SPVD in some regions, they often spread rapidly and are difficult to control, severely impacting sweet potato yield and quality. Bacterial wilt, primarily caused by *Ralstonia solanacearum*, is the main bacterial disease affecting sweet potato growth and development [74]. *Ralstonia solanacearum* is a typical soil-borne pathogen that infects a wide range of crops, including Solanaceae species and sweet potato [75]. In sweet potato, *Ralstonia solanacearum* infects the vascular system, impairing water and nutrient transport, which leads to wilting, chlorosis and ultimately plant death [74]. Furthermore, bacteria released from infected plants can further spread through irrigation water, rainfall, wounds and insect feeding. Therefore, heightened vigilance is also warranted against bacterial diseases.

Insect pests represent a pivotal biotic stress constraining sweet potato production, impacting multiple aboveground and underground plant organs. By feeding on leaves, storage roots, root systems and stems, these insect pests induce substantial yield reductions and impair the quality of sweet potato [76,77,78]. Among these, the sweet potato weevil (SPW; *Cylas formicarius*) is one of the most destructive pests globally, particularly in tropical and subtropical regions. It causes severe economic losses and is listed as a globally regulated quarantine pest [79]. The development of sweet potato cultivars resistant to SPW remains limited, and current management practices therefore rely on chemical pesticides, which pose serious environmental threats [78]. Wireworms (Coleoptera: Elateridae) pose another significant threat to sweet potato production, particularly in the southern United States, where they severely impact yield and quality [80,81]. In addition to sweet potato, wireworms infest a wide range of host crops, including wheat, maize and potato, facilitating their gradual spread to other regions as key pests limiting sweet potato cultivation.

These biotic factors do not work alone but often combine to create complex stresses, thereby greatly amplifying the damaging effects [50,52,62,63,82,83,84]. For instance, viruses are commonly transmitted to host plants via insect vectors. Aphids carrying SPFMV inoculate the virus into sweet potato plants during phloem-feeding, leading to systemic SPFMV infection of the host. Similarly, whiteflies frequently vector SPCSV, transmitting the pathogen to the host through feeding activity [53,60,85]. Importantly, once viral infection is established in sweet potato, it is virtually impossible to eradicate the virus through chemical intervention. Furthermore, mechanical damage and pest feeding activity create wounds in sweet potato, which serve as primary entry points for pathogens. For example, lesions generated by nematode infestation in sweet potato storage roots provide direct access for fungi and bacteria, resulting in the establishment of fungal and bacterial soft rot diseases. Such secondary infections further exacerbate storage root rot and reduce market value, and in extreme cases, lead to complete loss of edible and processing value [86,87]. These synergistic interactions make pest and disease management considerably more challenging, leading to greater yield losses than those caused by single stresses. However, the infection mechanisms employed by diverse pests and pathogens remain poorly understood [88]. Therefore, in-depth investigations into these mechanisms in sweet potato, as well as the elucidation of their interaction networks under complex stress conditions, are crucial for developing control strategies and enhancing the biotic stress resistance and yield stability of sweet potato.

### 2.2. Geographical Heterogeneity in Sweet Potato Pest and Disease Severity

Pests and diseases such as SPVD and SPW pose significant threats to sweet potato production. However, the severity of these biotic stresses exhibits considerable spatial heterogeneity due to climatic differences. Asia is the world’s primary sweet potato-producing region, where complex topography and diverse environments intensify pest and disease pressures and often involve interactions among multiple pathogens and pests. In addition, high temperatures and humidity across Asia favor the proliferation of these biotic threats [57,78,89]. For instance, there are distinct climatic variations between northern and southern China, which lead to substantial differences in the composition and damage severity of virus diseases [90]. The situation is more critical in Africa. Due to climate change, outdated technology, and improper management practices, losses caused by sweet potato pests and diseases are even more severe, posing a serious threat to food security. Among these biotic stresses, viruses have emerged as the primary factor constraining local sweet potato production [91,92,93,94,95,96,97]. By contrast, in developed countries, although pests and diseases continue to threaten sweet potato production, their impacts remain relatively manageable. This is largely due to the implementation of early warning systems and effective pest management practices [98].

### 2.3. The Spread of Pathogens and Insect Pests

The spread of sweet potato pathogens and insect pests involves a complex interplay among pathogens, insects, host plants, environmental conditions and human activities. Since sweet potato is an asexually propagated crop, its vegetative propagation makes it far more likely to carry pathogens or insect eggs compared to cereal crops, which facilitates the long-distance dispersal of pathogens and insect pests. The exchange of sweet potato germplasm and commercial trade have further promoted the spread of pathogens and insect pests to other producing areas [45,99]. Insects serve as critical vectors for disease transmission. Aphids are primary vectors of SPFMV. After feeding on infected plants, they acquire the virus within a short period and subsequently transmit it to healthy plants during feeding. The whitefly, as a key vector of SPCSV, has a longer retention period and higher transmission efficiency [100,101]. In addition, wounds inflicted by pests feeding on sweet potato storage roots and root systems provide entry points for fungal pathogens, indirectly facilitating the spread of soil-borne diseases [47].

The long-distance dispersal of pathogens and pests is further evidenced by numerous cross-regional records. SPCSV causes a severe disease and was first documented in Africa. Notably, this virus was first detected in the United States in 2007 [102]. Similarly, Sweet potato leaf curl virus (SPLCV) and the root-knot nematode were first documented in China in 2004 and 2013, respectively [103,104]. In addition, foot rot disease, caused by *Diaporthe destruens*, was identified in Zhejiang province of China, where it had not been previously reported, indicating its emergence as a new threat likely driven by pathogen spread [105]. Moreover, the threat posed by SPW is extending into higher latitudes, driven by global warming and agricultural migration [106,107]. Some viruses may also be transmitted through mechanical means, such as when contaminated farm tools introduce the virus into healthy plants via wounds. However, this mode of transmission is considered minor and incidental. In summary, the long-distance and cross-regional dispersal of sweet potato pathogens and insect pests is becoming increasingly prominent, exacerbated by global warming and agricultural practices. Together with potential mechanical transmission, these factors have led to more complex and widespread biotic stress risks, highlighting the necessity of enhanced surveillance and management for sustainable sweet potato production.

## 3. Sweet Potato Resistance Mechanisms Against Biotic Stresses: A Multidimensional Perspective

Sweet potato production is constantly threatened by a wide array of pathogens and insect pests. Therefore, elucidating the defense mechanisms of sweet potato in response to biotic stresses provides a theoretical foundation for breeding cultivars with enhanced stress resistance. However, due to the large and complex genome of hexaploid sweet potato (2*n* = B_1_B_1_B_2_B_2_B_2_B_2_ = 6x = 90), research progress on its biotic stress resistance has lagged significantly behind that in major cereal crops such as rice, wheat and maize [108]. Encouragingly, the completion of the sweet potato genome assembly and advances in omics technologies have laid a solid foundation for cloning genes associated with biotic stress responses. The past two years have witnessed important advances in deciphering resistance mechanisms against major pests and diseases, including SPW, *Fusarium* root rot, *Fusarium* wilt, *Rhizopus* soft rot and stem nematode disease, along with the identification of several functional genes with potential for resistance breeding. Collectively, recent advances have greatly enhanced our understanding of the mechanisms underlying both pathogen attack and host defense in sweet potato.

### 3.1. Advances in Sweet Potato Genomics Have Facilitated Research on Biotic Stress Resistance Mechanisms

The advancement of sweet potato genomics has been fundamental to elucidating the mechanisms underlying biotic stress resistance and identifying the associated genes. Initially, the completion of the diploid sweet potato genome sequence marked the beginning of the molecular era for this crop [109,110]. Subsequently, researchers assembled and refined the hexaploid sweet potato genome sequence, providing a high-quality genetic map for precisely identifying biotic stress resistance genes and elucidating their regulatory mechanisms [108,111,112]. Interestingly, in the process of dissecting the hexaploid sweet potato genome, researchers found that the ‘Tanzania’ genome contains 616 Toll-interleukin-1 receptor/nucleotide binding site/leucine-rich repeat (TIR-NBS-LRR) genes, which form high-density gene clusters on chromosomes [108]. TIR-NBS-LRR genes are core components of the plant immune system, mediating specific resistance against fungi, bacteria, viruses and insect pests [113,114,115]. Such retention likely enhances its adaptability to biotic stresses and provides key targets for breeding. Although remarkable progress has been made, the high heterozygosity of the sweet potato genome, in contrast to diploid plants such as rice and Arabidopsis, still imposes bottlenecks on the application of these large-scale genomic datasets. To further overcome research bottlenecks imposed by the complexity of polyploid genomes, researchers successfully assembled a complete, gap-free genome of the diploid wild sweet potato ‘Xiaoshu’. Xiaoshu has a homozygous genome, exhibits high self-compatibility and natural pollination, and is capable of forming storage roots. The completion of this high-quality genome facilitates the cloning and functional characterization of genes associated with biotic stress in sweet potato, thereby providing a superior platform for in-depth exploration of biotic stress resistance mechanisms [116]. Together, these breakthroughs in sweet potato genomics greatly promote the dissection of resistance mechanisms.

### 3.2. The Application of Omics Technologies in Sweet Potato

Omics technologies have provided essential tools for elucidating the mechanisms of sweet potato resistance to pathogens and insect pests. Beyond genomics, the application of transcriptomics, metabolomics and other omics approaches has enabled the multi-dimensional exploration of key genes and metabolites, offering new strategies for pest and disease control in sweet potato [117]. In this section, we focus on transcriptomics and metabolomics, which are commonly employed in studies of sweet potato resistance to pathogens and insect pests. For research progress in other omics technologies, readers are referred to the comprehensive review by Ahmed et al. [117].

Transcriptomics enables detection of dynamic gene expression changes in sweet potato under biotic stress and has become a widely used tool for deciphering defense signaling pathways. Unlike early transcriptomic applications in sweet potato under biotic stress [117,118,119], recent studies have placed greater emphasis on utilizing transcriptomics to evaluate expression differences between wild-type and transgenic plants (either overexpressing or knocking down resistance genes), thereby providing a more focused understanding of the regulatory mechanisms underlying resistance. Examples include studies on IbPIF1 (phytochrome-interacting factor 1, a transcription factor) and the IbCHYR1-IbZnFR complex (CHY zinc-finger and ring protein 1, an E3 ubiquitin ligase, and CCCH-type zinc-finger protein, a transcription factor). IbPIF1 is a key regulator of light signaling [120]. However, researchers have uncovered a novel role of PIFs in biotic stress responses. Transcriptomic analyses demonstrated that *IbPIF1* regulates the transcription of defense-related genes in sweet potato and is essential for resistance against stem nematodes [67]. Similarly, *IbCHYR1* and *IbZnFR* are indispensable for resistance to *Fusarium* root rot and *Fusarium* wilt in sweet potato [42]. In that study, transcriptomics not only revealed an association between *IbCHYR1* and root rot resistance but also identified downstream genes regulated by the IbCHYR1-IbZnFR complex. In summary, transcriptomics has advanced our understanding of sweet potato responses to biotic stress and has become an important tool for cloning genes associated with biotic stress resistance.

Metabolomics provides a complementary perspective by profiling metabolic changes associated with defense responses. For example, in sweet potato infected with *Ceratocystis fimbriata*, metabolomic analysis revealed dynamic changes in volatile organic compounds (VOCs) alongside alterations in key metabolites and defense-related enzyme activities. Notably, VOC profiles correlated significantly with these biochemical changes, offering a potential method for early disease monitoring [39]. In research on sweet potato resistance to SPW, integrated transcriptomic and metabolomic analyses revealed that genes involved in the shikimate pathway were induced by SPW infestation, accompanied by significant alterations in the levels of multiple downstream metabolites [78]. Therefore, the integrated analysis of metabolomics and transcriptomics enables the identification of gene-metabolite associations, offering a powerful strategy for elucidating the mechanisms of biotic stress resistance in sweet potato.

Emerging omics technologies, exemplified by single-cell and spatial transcriptomics, have well-documented advantages over conventional transcriptomic approaches in plant biotic stress research, yet remain severely underutilized in sweet potato studies. Unlike bulk transcriptomics, which generates averaged expression profiles from heterogeneous mixed tissue samples, these approaches enable high-resolution transcriptomic profiling for the dissection of biotic stress responses [121,122]. Specifically, single-cell transcriptomics enables the systematic elucidation of cell-type-specific defense responses and intercellular signaling communication during pathogen or pest infestation, while spatial transcriptomics retains the native in situ spatial context of gene expression to precisely map host–pathogen interaction dynamics at infection foci [122,123,124,125]. Despite these well-defined theoretical advantages, the practical implementation of these technologies in sweet potato research is currently hindered by multiple critical bottlenecks that directly limit their feasibility, including: (1) analytical complexity arising from the highly heterozygous hexaploid genome of sweet potato; (2) technical barriers to high-quality sequencing library construction from starch- and polyphenol-rich sweet potato tissues; and (3) the absence of validated species-specific cell marker databases for sweet potato. Successfully addressing these technical barriers will unlock the full theoretical potential of these technologies, and provide critical high-resolution molecular insights to advance the functional dissection of biotic stress resistance mechanisms and accelerate molecular resistance breeding in sweet potato.

### 3.3. Molecular Mechanisms of Biotic Stress Resistance

Driven by the decoding of the sweet potato genome and advances in biotechnology, remarkable progress has been made over the past two years in understanding the responses of sweet potato to pathogens and insect pests (Table A2). In this section, we summarize and discuss key recent studies in this area, and propose a series of perspectives and conceptual frameworks to provide novel insights for future research on disease and pest resistance in sweet potato.

**Figure 2:**
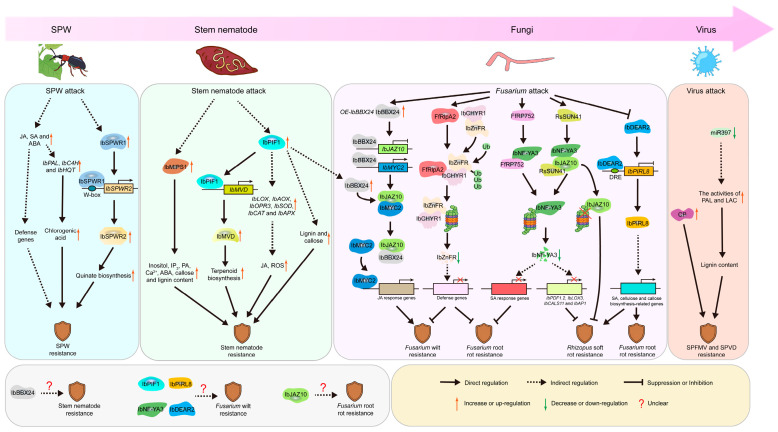
Summary of resistance mechanisms against major pathogens and insect pests (SPW, stem nematode, fungi and viruses) in sweet potato. SPW: upon SPW feeding, the expression levels of a series of downstream genes (including *IbSPWR1*, *IbPAL*, *IbC4H*, *IbHQT* and other defense genes) are induced. This upregulation promotes the biosynthesis of chlorogenic acid and quinate, thereby enhancing sweet potato resistance to SPW. Stem nematode: upon stem nematode attack, the expression of *IbMIPS1* and *IbPIF1* is upregulated. IbMIPS1 increases the contents of inositol, IP_3_, PA, Ca^2+^, ABA, callose and lignin, thereby enhancing resistance to stem nematode. IbPIF1, acting as a transcription factor, directly upregulates *IbMVD* expression, leading to increased terpenoid biosynthesis. In addition, IbPIF1 indirectly promotes the upregulation of JA- and ROS-related genes, as well as the biosynthesis of lignin and callose. Fungi: In *IbBBX24* overexpressing plants, IbBBX24 suppresses *IbJAZ10* expression while promoting *IbMYC2* expression. It also disrupts the binding of IbJAZ10 to IbMYC2, thereby relieving the repression of JA-responsive genes and enhancing resistance to *Fusarium* wilt. The effectors FfRlpA2 and FfRP752 hijack IbCHYR1 and IbNF-YA3, respectively, promoting IbZnFR and IbNF-YA3 degradation and thereby suppressing the expression of downstream defense genes. This leads to compromised resistance to *Fusarium* wilt, root rot and soft rot, respectively. Similarly, another effector, RsSUN41, employs a similar mechanism, promoting IbNF-YA3 degradation, resulting in suppressed resistance to soft rot. Upon infection, the expression increase of *IbPIRL8* is mediated by the direct binding of IbDEAR2 to its promoter. The subsequent accumulation of IbPIRL8 protein then initiates multiple defense pathways, ultimately conferring resistance to soft rot and root rot. Viruses: The mechanisms underlying viral infection and resistance in sweet potato remain largely unclear. Several studies have shown that expression of the viral CP gene or STTM-mediated downregulation of *miR397* levels can enhance resistance to viral diseases such as SPFMV and SPVD in sweet potato.

Although the SPW is one of the most significant pests affecting sweet potato and poses a major threat to sustainable production, the mechanisms underlying plant defense against SPW had long remained unclear. Early studies focused on metabolic and hormonal responses. SPW infestation induces the accumulation of jasmonic acid (JA), salicylic acid (SA) and abscisic acid (ABA), key regulators of plant immunity, which in turn promote the expression of downstream defense-related genes [126,127,128,129,130]. These hormones were also shown to activate chlorogenic acid biosynthesis-related genes such as *IbPAL*, *IbC4H* and *IbHQT*, leading to the accumulation of chlorogenic acid, a metabolite associated with SPW resistance [131]. Additionally, transcriptomic analysis was conducted using the resistant cultivar Kyushu No. 166 (K166) and the susceptible cultivar Tamayutaka. The results revealed that genes upregulated in the resistant cultivar are primarily involved in phosphorylation, metabolic pathways and terpenoid biosynthesis [132]. Although these studies have advanced our understanding, the key genes conferring resistance to SPW have not been cloned. A breakthrough has addressed this gap. SPW infestation induces the expression of *IbSPWR1* (*SPW resistance 1*), which encodes a WRKY transcription factor. IbSPWR1 specifically binds to the W-box element in the promoter of *IbSPWR2* (*SPW resistance 2*), which encodes a dehydroquinate synthase, thereby regulating the shikimate-quinate metabolic pathway and promoting quinate biosynthesis (Figure 2). Quinate confers sweet potato resistance to SPW by directly inhibiting SPW feeding behavior and disrupting its digestive enzyme activity [78]. Together, these findings elucidate the molecular mechanism underlying sweet potato defense against SPW infestation and provide potential genes for breeding.

Substantial advances have also been achieved in elucidating the defense mechanisms of sweet potato against stem nematodes. Previous research on stem nematodes in sweet potato was largely confined to cytological observations and phytopathological studies [68,133,134], and the molecular mechanisms underlying sweet potato stem nematode resistance remain poorly understood. A study revealed that overexpression of *IbMIPS1* increased the levels of inositol, IP_3_ (inositol trisphosphate), PA (phosphatidic acid), Ca^2+^, ABA, callose and lignin (Figure 2) [21]. These components are critical for sweet potato defense against stem nematodes, as callose and lignin reinforce cell wall integrity to block nematode penetration, while Ca^2+^ and ABA trigger downstream immune signaling cascades that inhibit nematode reproduction. Recent studies have revealed that the expression of *IbPIF1* is strongly induced in sweet potato upon stem nematode infestation. IbPIF1 directly binds to the promoter of *IbMVD* (a pivotal gene in the regulation of terpenoid synthesis), inducing its expression and thereby modulating terpenoid-mediated resistance (Figure 2) [67,135]. Terpenoids are known to play a critical role in plant defense against biotic stresses by disrupting the nematode nervous system [136]. Consequently, this regulatory module governs sweet potato resistance to stem nematodes.

Multiple critical advances have also been made in elucidating the molecular mechanisms underlying sweet potato resistance to pathogens. Overexpression of *IbBBX24* (encoding a B-box transcription factor) promotes JA accumulation, whereas its silencing has the opposite effect. IbBBX24 represses expression of *IbJAZ10* (encoding a jasmonate-ZIM domain protein) while activating *IbMYC2* (encoding a bHLH transcription factor), and competes with IbMYC2 for binding to IbJAZ10, thereby relieving IbMYC2 from suppression and activating downstream JA-responsive genes. This cascade confers resistance to *Fusarium* wilt (Figure 2) [137]. A mechanism commonly employed by pathogens is the secretion of effector proteins that target and hijack host proteins to facilitate invasion. In sweet potato, three effectors (FfRlpA2, FfRP752 and RsSUN41) have recently been shown to hijack host proteins, thereby facilitating infection and disease development. The *Fusarium* effector FfRlpA2 targets the host IbCHYR1 protein, stabilizing it and promoting its interaction with IbZnFR. This leads to the degradation of IbZnFR, a positive regulator of resistance, thereby suppressing host defense and facilitating infection (Figure 2) [42]. Another study revealed that the effector FfRP752 from *Fusarium foetens* f. sp. *batatas* DX94 hijacks IbNF-YA3 (encoding a nuclear factor-YA transcription factor) to promote infection. Conversely, overexpression of *IbNF-YA3* enhances root rot resistance as IbNF-YA3 directly activates the transcription of the SA-mediated signaling pathway that is essential for plant immunity [138]. Recent work has also identified a similar mechanism involving IbJAZ10 and IbNF-YA3 in regulating soft rot resistance. In resistant varieties, the *Rhizopus stolonifer* MY03 effector RsSUN41 hijacks IbJAZ10 to degrade IbNF-YA3 [139]. Elevated expression levels of *IbNF-YA3* enhance resistance to soft rot, indicating that IbNF-YA3 acts as a positive regulator of soft rot resistance. Taken together, these findings reveal a conserved mechanism in sweet potato: phytopathogen effectors universally target and hijack host defense-related proteins to suppress host immune responses.

Research on sweet potato resistance mechanisms against viral diseases remains in the early stages. Within this context, multiple experimentally validated findings have been reported to advance this field. First, several studies have utilized transcriptomic approaches and established systems for resistance identification and classification of sweet potato against viral diseases, laying the foundation for understanding host responses to SPVD [56,140]. Second, transgenic approaches introducing exogenous genes such as *coat protein* (*CP*) into sweet potato have been experimentally confirmed to confer enhanced resistance against SPFMV, SPCSV, SPVG and sweet potato mild mottle virus (SPMMV) [51,141,142]. Third, a recent study employed short tandem target mimic (STTM) technology to suppress the expression of *miR397*, leading to increased activities of phenylalanine ammonia-lyase (PAL) and laccase (LAC), thereby promoting lignin biosynthesis and enhancing resistance to SPVD in transgenic plants by lignin reinforcement of the cell wall barrier to prevent viral entry [143]. Despite these advances, the molecular mechanisms underlying sweet potato responses to viral infection and the basis of resistance remain elusive. It is difficult to identify anti-viral genes through traditional phenotypic identification and gene mapping in sweet potato, thus underscoring the urgent need for a breakthrough in this field. Looking forward, to bridge the aforementioned knowledge gap and elucidate the mechanisms underlying sweet potato responses to viral infection, we propose two priority prospective research strategies beyond conventional approaches. (1) Leveraging AI (such as the AlphaFold tool) to dissect viral protein structures and predict their potential interacting proteins in sweet potato may serve as a highly efficient strategy for cloning resistance genes. (2) Integrating emerging high-resolution omics tools, particularly single-cell and spatial transcriptomics, to pinpoint candidate resistance genes at cellular and spatial dimensions represents another valuable approach deserving further investigation.

### 3.4. Proposed Working Hypotheses and Future Research Perspectives

Based on the aforementioned findings on the molecular defense mechanisms of sweet potato against biotic stresses, we here propose a series of testable working hypotheses and highlight key unexplored research directions (Table A3). (1) The expression levels of *IbBBX24* are also significantly altered in *IbPIF1* transgenic lines [67]. Given that *IbBBX24* is known to regulate *Fusarium* wilt resistance [137], this suggests that *IbBBX24* may also be involved in regulating sweet potato resistance to stem nematodes and could be directly regulated by *IbPIF1* (Figure 2). This hypothesis represents a promising, unexplored avenue for future functional research. (2) At the molecular level, the resistance genes involved in the aforementioned studies (*IbBBX24*, *IbPIF1*, *IbJAZ10* and *IbNF-YA3*) appear to interconnect and may function within a common pathway (Figure 2). (3) At the functional level, IbJAZ10 participates in defense against both *Fusarium* wilt and soft rot, while IbNF-YA3 is involved in resistance to soft rot and root rot. Similar to *IbNF-YA3*, new findings have revealed that overexpression of *IbPIRL8* (encoding a Ras-group related leucine-rich repeat protein) confers resistance to soft rot and root rot in sweet potato [144]. Considering the potential conservation of these functions (Figure 2), we hypothesize that these regulators may have additional functions: IbJAZ10 might also participate in root rot resistance, while IbNF-YA3 and IbPIRL8 might play roles in defense against *Fusarium* wilt. Interestingly, the downstream genes regulated by these pathways are primarily implicated in terpenoid biosynthesis, JA signaling, callose deposition and the reactive oxygen species (ROS) burst, which are integral to immune responses against a broad spectrum of pathogens and insect pests [145,146,147]. Therefore, whether the regulation of biotic stress responses by *IbBBX24*, *IbPIF1*, *IbJAZ10*, *IbNF-YA3* and *IbPIRL8* is broad-spectrum represents a valuable direction for future research.

## 4. Stress Synergy and Defense Regulation in Sweet Potato

**Figure 3:**
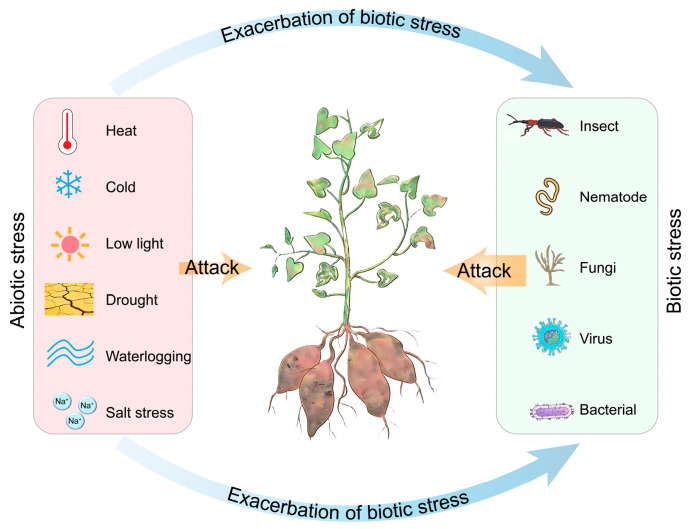
Abiotic stresses can exacerbate the damage caused by biotic stresses to sweet potato. Abiotic stress factors (e.g., heat, cold, low light, drought, waterlogging and salt stress) influence the spread, feeding behavior, and reproduction of insect pests and pathogens, thereby exerting synergistic effects that ultimately compromise storage root growth, quality and yield.

Currently, the combined effects of abiotic and biotic stresses have emerged as a key constraint on sustainable sweet potato production. Compared with individual stresses, their synergistic effects pose a more severe threat to sweet potato growth. Significant progress has been made in understanding the combined effects of biotic and abiotic stresses on crop growth and development in cereal crops [23,148,149]. For instance, prolonged low-light conditions drastically increase the susceptibility of rice plants to *Magnaporthe oryzae*, the causal agent of rice blast disease [150]. In contrast to cereal crops, considerable knowledge gaps remain in sweet potato. Driven by global climate change, abiotic stresses have significantly reshaped the geographical distribution and spread patterns of major pathogens and insect pests in sweet potato. Specifically, several studies have revealed that the geographical distribution of destructive sweet potato pests such as weevils and whiteflies is no longer constrained by historical temperature thresholds owing to climate warming, thereby facilitating their population expansion into higher latitudes and altitudes [151,152]. Furthermore, experimental evidence from sweet potato indicates that drought stress increases the feeding activity of root-knot nematodes, while humidity levels influence whitefly reproduction [153,154]. Taken together, it can be reasonably inferred from the combination of these lines of evidence that the synergistic effects of abiotic and biotic stresses will continue to intensify the threat of pathogens and insect pests to sweet potato production (Figure 3) [155,156]. Thus, further investigations on relevant mechanisms are urgently required. Multiple studies in sweet potato have preliminarily identified several genes potentially involved in the crosstalk between biotic and abiotic stress responses. Mitogen-activated protein kinases (MAPKs) are essential for various signaling pathways triggered by both abiotic and biotic stresses [157,158,159]. In sweet potato, the MAPK family genes *IbMPK3* and *IbMPK6* are induced by multiple abiotic stresses, including salt and cold. Additionally, IbMPK3 and IbMPK6 positively regulate bacterial pathogen resistance and pathogenesis-related (PR) gene expression [160]. Similarly, the expression of the sweet potato *IbMIPS1* (a key rate-limiting enzyme in myo-inositol biosynthesis) is induced by both biotic and abiotic stresses, including salt, drought, ABA and stem nematode infestation. Moreover, overexpression of *IbMIPS1* significantly enhances resistance to stem nematodes, and improves salt and drought tolerance [21]. Given that *IbMPK3*, *IbMPK6* and *IbMIPS1* mediate responses to both biotic and abiotic stresses, we hypothesize that regulatory interconnections exist among these genes. Notably, a previous study reported that IbMPK3 and IbMPK6 mediate the phosphorylation of IbSPF1 (an SP8-binding factor) to modulate bacterial resistance in sweet potato [161]. Based on this finding, we speculate that IbMPK3 and IbMPK6 may physically interact with and phosphorylate IbMIPS1, thereby serving as a mechanistic bridge mediating the crosstalk between biotic and abiotic stress signaling pathways. However, it is important to recognize that despite notable progress in identifying candidate genes implicated in stress signal integration, the precise mechanisms underlying crosstalk between biotic and abiotic stress responses in sweet potato remain largely unresolved. Experimental validation of this proposed interaction in sweet potato would substantially advance our understanding of stress signal integration in this crop.

## 5. Strategies for Sustainable Sweet Potato Production

To address the diverse biotic stresses threatening sweet potato production, several proactive strategies are essential. In this section, we discuss three complementary approaches: the establishment of early warning systems for pests and diseases, the optimization of cultivation practices (including crop rotation, use of virus-free seedlings, and soil microbiota management), and the genetic improvement of pest and disease resistance through molecular breeding and emerging biotechnologies. Collectively, these strategies aim to reduce yield losses, minimize chemical inputs, and promote the long-term sustainability of sweet potato production.

### 5.1. Establishment of Early Warning Systems for Pest and Disease Management

In managing pathogens and insect pests in sweet potato, a prevention-centered control system serves as the cornerstone of sustainable production. This system not only effectively manages pests and diseases but also safeguards the ecological environment. Moreover, as climate change intensifies and continuously alters the geographical range of pathogen and insect pest outbreaks, the establishment of early warning systems has become increasingly critical. An early warning system relies on a precision monitoring network that integrates multiple technologies, including remote sensing, meteorological data, the Internet of Things (IoT), big data and AI. These technologies enable early detection, dynamic tracking and prediction of pests and diseases, thereby providing a basis for timely intervention [162,163]. Complementing these macroscopic monitoring approaches, the detection of potential pathogens using molecular markers represents another core strategy for disease early warning. For example, researchers have developed a specific molecular marker for DX94, termed FfIdnDH, which can serve as a species-specific detection tool for *F. foetens*, facilitating the monitoring and early warning of root rot pathogens in the field [138].

The application of early warning systems for sweet potato not only facilitates the transition from ‘passive response’ to a ‘proactive prediction’ model but also enhances both the quality and efficiency of sweet potato production. On the one hand, early warning systems enable the forecasting of pest and disease risks, guiding farmers to implement targeted controls and thereby minimize the indiscriminate use of pesticides. This helps reduce chemical residues and contamination of soil and water. On the other hand, such precision management effectively suppresses the spread of pests and diseases, reducing yield losses and control costs while simultaneously improving the quality and market value of sweet potato products. Although early warning strategies currently face challenges such as regional disparities in technological infrastructure and high equipment operation and maintenance costs, their significant technical and economic advantages, along with the potential for cost reduction through technological and equipment improvements, make early warning systems a key investment priority for advancing the sustainable development of the sweet potato industry.

### 5.2. Optimizing Cultivation Practices for Sweet Potato

Proper field management practices are essential for mitigating biotic stresses in sweet potato by creating a field environment that simultaneously suppresses pests and promotes robust plant growth. However, continuous cropping leads to the progressive accumulation of pathogens and insect pests, thereby aggravating biotic stress damage. In addition, long-term continuous cropping causes soil nutrient imbalances and the deterioration of soil physicochemical properties, further compromising the biotic stress tolerance of sweet potato plants [38,164]. Rational crop rotation effectively alleviates these adverse effects. As well-established and highly effective cultural strategies, crop rotation reduces damage from biotic stress by modifying the spatial and temporal layout of crops [165,166,167]. On the one hand, rotation with other crops deprives sweet potato-specific pathogens, nematodes and insect eggs of suitable hosts, thereby suppressing their survival and significantly reducing population densities in the soil. For instance, rotating sweet potato with maize, wheat, rice or soybean significantly reduces pathogen and insect pest pressure, and lowers the incidence and severity of the corresponding diseases. On the other hand, crop rotation optimizes soil physicochemical properties, enhances soil fertility and promotes robust root development in sweet potato, thus enhancing its resistance to biotic stresses [168].

In addition to optimized cropping patterns, the production and use of virus-free sweet potato seedlings also demand attention. Viral diseases represent one of the major biotic constraints on yield and quality in sweet potato production. Once plants are infected, viruses are difficult to eradicate by chemical control methods. Therefore, the production and use of virus-free sweet potato seedlings have emerged as the primary strategy for viral disease management. Virus-free sweet potato seedlings are produced through an integrated process involving tissue culture, virus detection and elimination. These virus-free seedlings enable elite cultivars to realize their full genetic potential, an advantage that is especially pronounced in high-yielding varieties susceptible to viral infection. Although techniques for producing virus-free seedlings are now well-established, their application faces multiple challenges. The application of virus-free seedlings increases production costs, limiting their acceptance and utilization among farmers, particularly in developing regions such as Africa. Furthermore, the risk of post-planting reinfection remains a major unaddressed challenge in field application [169]. The virus-free seedlings may rapidly become reinfected with viruses within a single growing season via aphids and whiteflies, mechanical transmission during agricultural operations and virus-contaminated field soils, drastically diminishing the yield benefits of virus-free materials and reducing farmers’ willingness to adopt them long-term. Moreover, the global absence of an authoritative certification framework, insufficient detection technologies for emerging and latent viruses, and high infrastructural and technical requirements collectively constrain the widespread application of virus-free sweet potato seedlings. Therefore, future efforts need to focus on reducing production costs, developing more reliable and low-cost virus detection technologies, and establishing a global certification framework, which are crucial for promoting the application of virus-free sweet potato seedlings.

Modulating the community structure of beneficial soil microbiota and enriching their populations represent another critical approach to mitigating the impacts of biotic stresses on sweet potato. A well-structured soil microbiome enhances sweet potato resistance to biotic stresses. This occurs through the direct suppression of pathogens by beneficial microorganisms and their promotion of plant growth [168,170,171]. Despite these promising advantages, microbiota-mediated strategies still face prominent practical limitations in field application. Specifically, their insufficient field stability, distinct site specificity driven by native soil background differences, and insufficient effectiveness across diverse climatic and edaphic conditions greatly restrict their application [172]. The colonization persistence of introduced beneficial microbes is easily disturbed by regional soil properties and seasonal environmental variations, which further exacerbates the unstable control efficacy across different planting regions. Therefore, systematic exploration of adaptive regulation strategies for microbiota is essential to overcome these challenges in future studies. In summary, optimized cropping patterns, the use of virus-free seedlings and modulation of soil microbiota collectively mitigate the adverse effects of various biotic stresses on sweet potato.

### 5.3. Genetic Improvement of Pathogen and Insect Pest Resistance in Sweet Potato Cultivars

In response to the rising incidence of pathogens and insect pests, improving sweet potato cultivars for enhanced resistance has become a critical and economic measure to ensure sweet potato production. Historically, conventional cross-breeding has been the dominant strategy in sweet potato breeding, and a number of cultivars with high pathogen and insect pest resistance have been successfully developed. However, due to the complex genome structure of sweet potato, unstable trait segregation in hybrid progeny and long breeding cycles, developing new cultivars with high resistance to pathogens and insect pests through conventional breeding has become increasingly challenging. Approaches such as molecular marker detection, cloning of resistance genes and targeted gene editing enable precise selection and improvement of traits, thereby offering distinct advantages such as high efficiency, shorter breeding cycles, and precise targeting [173,174,175,176]. Furthermore, significant breakthroughs in emerging biotechnologies (such as AI and synthetic biology) have offered novel pathways to achieve precise and efficient improvement in sweet potato breeding [177,178,179].

Molecular markers have been widely utilized in breeding. Through approaches such as traditional quantitative trait loci (QTL) mapping and genome-wide association studies (GWAS), researchers have identified numerous genetic loci associated with pest and disease resistance and developed corresponding molecular markers, thereby providing essential tools for sweet potato resistance breeding [180]. In recent years, several key genes mediating sweet potato responses to biotic stresses have been successfully cloned (Figure 2 and Table A2). Resistance genes such as *IbJAZ10*, *IbCHYR1*, *IbSPWR1* and *IbSPWR2*, derived from natural variation, show great potential in cultivar improvement [42,78,139]. Based on these findings, developing molecular markers for cloned resistance genes enables their rapid detection in cultivars or hybrid progeny. The cloning of resistance genes and development of molecular markers both rely on the systematic exploration and conservation of sweet potato germplasm resources. However, current efforts in the collection, evaluation and utilization of germplasm resources remain insufficient, leaving many potential resistance genes underexplored. It is important to note that the co-evolution between plants and pests means that reliance on a single resistance gene often leads to weakened or breakdown resistance [181,182]. To diversify the genetic basis of resistance in breeding, future efforts should focus on strengthening the collection, conservation, utilization and precise phenotypic characterization of sweet potato germplasm resources, thereby providing new key genes and resources for breeding.

Gene editing technology, known for its precision and efficiency, has emerged as one of the most promising tools in sweet potato molecular breeding. However, the application of gene editing technologies, particularly CRISPR (Clustered Regularly Interspaced Short Palindromic Repeats), to elucidate biotic stress defense mechanisms and enhance breeding in sweet potato remains limited due to several challenges. These include off-target effects, difficulties in genetic transformation, and the complexity of simultaneously editing multiple gene copies and generating homozygous genotypes in sweet potato. Given these constraints, RNAi, a more accessible alternative, has been more extensively applied in functional studies of genes related to biotic stress resistance in sweet potato [42,67,78,137,138,139,144,183]. The ability of RNAi to concurrently silence multiple alleles may account for its broader adoption. Despite these challenges, the significant advantages of gene editing underscore its vast potential, motivating future efforts to develop more efficient editing tools and improve genetic transformation efficiency for creating novel cultivars. Encouragingly, multiple sweet potato transformation systems have recently been established [184,185]. Furthermore, CRISPR/Cas9 technology has been successfully employed in sweet potato to edit key genes, including *IbPDS*, *IbGBSSI*, *IbSBEII* and *IbMYB308L*, thereby enhancing quality traits and enabling the creation of albino seedlings for functional studies [186,187,188,189]. Collectively, these advances demonstrate the feasibility of gene editing tools in sweet potato, laying a solid foundation for applying gene editing technology to enhance biotic stress resistance breeding in sweet potato.

Synthetic biology allows for precise trait modification and the introduction of novel resistance traits, complementing traditional breeding in sweet potato. For instance, given that quinate enhances sweet potato resistance to SPW, engineering the quinate biosynthesis pathway represents a feasible technical route, in addition to targeting *IbSPWR1* and *IbSPWR2*. Currently, although the application of synthetic biology in sweet potato is limited, its successful implementation in crops such as rice suggests that its use in sweet potato breeding to enhance resistance warrants further investigation [190,191,192].

In parallel with these biotechnological advances, AI has emerged as a transformative force for accelerating crop breeding. AI can efficiently process the massive datasets involved in crop breeding, including genomic, phenotypic and environmental data, thereby enabling intelligent and precise breeding that reduces costs and improves efficiency. Traditional phenotypic evaluation for pest and disease resistance in sweet potato relies on manual measurement, which suffers from low efficiency and subjectivity. Through advanced imaging technologies, AI enables high-throughput and accurate phenotypic identification [193,194]. Moreover, AI for protein structure prediction, exemplified by AlphaFold, has been widely applied in both plants and animals [195,196]. This tool can predict the structures of effector proteins of pathogens, revealing their vulnerabilities. AlphaFold can also predict the structures of sweet potato proteins, thereby facilitating the functional analysis of resistance genes, and enable the design of gene editing targets based on protein structure for gene modification. Recently, researchers have proposed an AI model that integrates and analyzes vast amounts of data to generate optimized breeding strategies [197]. This approach transforms crop breeding from traditional methods to precise design breeding. Furthermore, AI can also replace manual labor in conventional cross-breeding, thereby accelerating the breeding cycle [198]. In summary, AI holds great potential for applications in phenotypic evaluation, gene functional studies and breeding practice, which can accelerate the breeding process.

## 6. Challenges and Perspectives

Establishing early warning systems, promoting virus-free seedlings, improving cultivation practices and modulating soil microbiota are important strategies for achieving sustainable sweet potato production. Significant progress has been made in these areas in recent years. However, the high cost associated with some of these practices currently limits their widespread adoption in developing regions. Thus, technological innovations are needed to reduce these costs.

Recent studies have provided insights into the mechanisms underlying sweet potato biotic stress responses, but these processes remain largely elusive. Additional efforts are needed to clone resistance genes and elucidate their functions (Table A3). This is particularly critical for viral diseases, which pose a serious threat to sweet potato production. Given that phenotypic identification techniques have become more advanced and accurate, and that new technologies such as emerging omics technologies (such as single-cell and spatial transcriptomics), biotechnological tools, bioinformatics and AI are being applied, these developments offer the possibility for researchers to unravel the mechanisms of sweet potato resistance to viruses and to breed resistant varieties. Future research should prioritize understanding viral infection mechanisms and identifying corresponding resistance genes.

Sweet potato production is threatened by both biotic and abiotic stresses, yet the molecular mechanisms underlying the combined effects of these stresses remain largely unknown. For instance, despite their known roles in biotic and abiotic stress responses, it remains unclear whether *IbMPK3*, *IbMPK6*, and *IbMIPS1* play pivotal roles in the crosstalk between biotic and abiotic stress responses [21,160]. Future research should increase attention on elucidating the mechanisms underlying the interactive effects of biotic and abiotic stresses on sweet potato production. This will provide a theoretical foundation for the genetic improvement of biotic stress resistance in sweet potato.

Conventional omics technologies have been applied in research on sweet potato biotic stress. Nevertheless, the application of emerging technologies such as single-cell and spatial transcriptomics faces substantial challenges. These emerging techniques offer a level of resolution unattainable with traditional approaches, enabling the precise analysis of gene expression within individual cells and across different spatial locations in plant tissues under complex stresses [199]. Future work should therefore strengthen efforts to apply single-cell transcriptomics, spatial transcriptomics and other novel omics approaches in sweet potato biotic stress studies to characterize the responses of different cell types to biotic stress.

Sweet potato germplasm resources harbor important pathogen and insect pest resistance genes and are fundamental for sweet potato breeding. However, the collection, evaluation, utilization and conservation of sweet potato germplasm resources remain insufficient, and many valuable resistance genes are yet to be discovered and functionally characterized. Unlike seed-propagated crops such as rice and wheat, the long-term conservation of sweet potato germplasm resources presents significant challenges [200]. Therefore, strengthened efforts in the collection, evaluation, utilization, and long-term conservation of sweet potato germplasm are essential.

Gene editing technology, with its high precision and ability to generate stably inherited genetic variations, offers a novel approach for improving biotic stress resistance in sweet potato. However, the application of this technology in sweet potato still faces multiple challenges, limiting its adoption. Future efforts should focus on developing more efficient gene editing tools and further improving genetic transformation efficiency to advance the application of gene editing in sweet potato. In addition to advancing gene editing, emerging technologies such as AI and synthetic biology hold significant potential in breeding and warrant further in-depth investigation.

To address these challenges and more effectively carry out the necessary work, we propose a roadmap for future research. In the short term (1–3 years), efforts will focus on foundational research, including systematic phenotypic and genotypic evaluation of core sweet potato germplasm for resistance to major biotic stresses, as well as the establishment of an open-access germplasm database. In the medium term (4–8 years), research priorities will shift toward mechanistic elucidation and technological system development. Key works include dissecting core regulatory networks, establishing single-cell and spatial transcriptomics platforms for sweet potato, optimizing gene editing systems and the production of virus-free seedlings, and developing an AI-based high-throughput phenotyping platform for pest and disease monitoring. In the long term (8–10 years), the focus will be on breeding applications and the construction of a global sustainable production system. This entails developing multi-resistant sweet potato cultivars that combine high yield, superior quality and adaptability to marginal lands, establishing a global pest and disease monitoring and early warning system, and creating an AI-assisted design system to shift sweet potato breeding from empirical to precision-based approaches. This roadmap closely aligns fundamental research with the needs of the sustainable production of sweet potato. Its phased implementation is expected to relieve current bottlenecks in biotic stress research and unlock the crop’s potential to contribute to global food security, particularly in developing regions.

## 7. Conclusions

**Figure 4:**
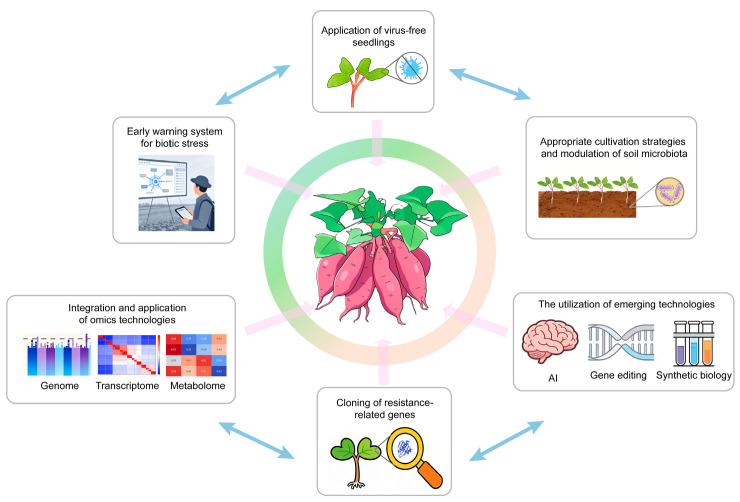
Multiple strategies to enhance sweet potato resistance to biotic stresses. From a cultivation perspective, sweet potato growth can be ensured through establishing early warning systems for biotic stresses, applying virus-free seedlings, adopting appropriate cultivation strategies, and modulating soil microbiota. From a breeding perspective, the integration of omics technologies, the utilization of emerging technologies (such as gene editing, AI and synthetic biology), and the cloning of resistance-related genes can provide both genetic resources and a theoretical basis of molecular mechanisms for breeding aimed at enhancing biotic stress resistance. Collectively, these strategies enhance the ability of sweet potato to respond to biotic stresses, thereby safeguarding food security.

Sweet potato holds an irreplaceable value for global food security, owing to its high yield and remarkable adaptability, which enables normal growth even in marginal or adverse environments. However, as highlighted in this review, sweet potato production is under severe threat from various biotic stresses, a threat that is being intensified by climate change. To date, significant progress has been made in the cloning of biotic stress resistance genes in sweet potato (Table A2). Furthermore, the assembly of the high-quality sweet potato genomes and application of multi-omics technologies have further facilitated the identification of key resistance genes and the elucidation of their defense mechanisms. These advances have substantially enhanced our understanding of the mechanisms underlying sweet potato responses to biotic stresses, and have provided novel resistance gene resources for breeding. In addition, the establishment of early warning systems for pathogens and insect pests, the application of virus-free seedlings, the improvement of cultivation strategies, and the utilization of emerging technologies such as AI, single-cell transcriptomics and gene editing hold great significance for addressing these challenges. These efforts are crucial for enhancing biotic stress resistance in sweet potato and promoting its sustainable production (Figure 4 and Table A4).

## Data Availability

No new data were created or analyzed in this study.

## Abbreviations

The following abbreviations are used in this manuscript:

ABA	Abscisic acid
AI	Artificial intelligence
CP	Coat protein
FAO	Food and Agriculture Organization of the United Nations
GWAS	Genome-wide association studies
IoT	Internet of Things
IP3	Inositol trisphosphate
JA	Jasmonic acid
LAC	Laccase
MAPKs	Mitogen-activated protein kinases
PA	Phosphatidic acid
PR gene	Pathogenesis-related gene
QTL	Quantitative trait loci
RNAi	RNA interference
RNA-seq	RNA sequencing
ROS	Reactive oxygen species
SA	Salicylic acid
SNP	Single nucleotide polymorphism
SPCSV	Sweet potato chlorotic stunt virus
SPFMV	Sweet potato feathery mottle virus
SPLCV	Sweet potato leaf curl virus
SPMMV	Sweet potato mild mottle virus
SPVD	Sweet potato virus disease
SPVG	Sweet potato virus G
SPW	Sweet potato weevil
STTM	Short tandem target mimic
TIR-NBS-LRR	Toll-interleukin-1 receptor/nucleotide binding site/leucine-rich repeat
VOCs	Volatile organic compounds

## Appendix A

**Table A1:** Biological characteristics and yield damage of primary fungal, viral, nematode, bacterial and insect pests in sweet potato.

Type of Disease or Pest	Causal Agent or Taxonomic Classification	Symptoms	Primary Transmission Route	Geographic Hotspot	Yield Loss
Fungi, *Fusarium* root rot	*Fusarium solani* f. sp. *batatas*	Root necrosis, plant stunting and chlorosis	Soil, long-distance dissemination via infected storage roots/seedlings	Globally distributed	30% in general fields, and complete crop failure in severely infested fields
Fungi, *Fusarium* wilt	*Fusarium oxysporum* f. sp. *batatas*	Wilting of leaves and vascular browning of vines	Soil, long-distance dissemination via infected storage roots/seedlings	Globally distributed	10–50%
Fungi, black rot	*Ceratocystis fimbriata* f. sp. *batatas*	Black, firm and sunken lesions on the storage root surface	Infected storage roots and seedlings	Globally distributed	At least 10%
Fungi, soft rot	*Rhizopus stolonifer*	Rapid softening and maceration of storage roots following wound infection, with decay of plant tissue accompanied by fungal growth	Wound infection	Globally distributed	~15%
Virus, SPVD	SPFMV + SPCSV	Extreme plant stunting, leaf chlorosis, curl and distortion, and drastically reduced storage root size and malformation	Aphids, whiteflies and infected storage roots	Globally distributed, especially in Africa	Up to 90%
Virus, SPFMV disease	SPFMV	Foliar chlorotic mottle and purple ring spots	Aphids and infected storage roots	Globally distributed	~10% under single infection
Virus, SPCSV disease	SPCSV	Mild chlorosis and stunting	Whiteflies and infected storage roots	Globally distributed	~10% under single infection
Nematode, southern root-knot nematode	*Meloidogyne incognita*	Plant stunting, chlorosis, defoliation, and total plant death, formation of characteristic root galls on fibrous roots	Soil, long-distance dissemination via infected storage roots/seedlings	Globally distributed, especially in Africa and southern China	5–30%
Nematode, stem nematode	*Ditylenchus destructor*	Stem rot, brown discoloration and spongy cavity rot inside storage roots	Soil, long-distance dissemination via infected storage roots/seedlings	Globally distributed, especially in Asia	20–50%
Nematode, reniform nematode	*Rotylenchulus reniformis*	Root browning and rot and plant chlorosis, stunting	Soil, long-distance dissemination via infected storage roots/seedlings	Globally distributed, especially in Africa, southern China	~10%
Bacteria, Bacterial wilt	*Ralstonia solanacearum*	Wilting, chlorosis, vascular browning and ultimately plant death	Soil, long-distance dissemination via infected storage roots/seedlings	Globally distributed, especially in Asia and Africa	>80% loss or complete crop failure in severely infested fields
Bacteria, Bacterial soft rot	*Pectobacterium* *carotovorum*	Rapid softening and slimy maceration	Wound infection and overwintering in soil	Globally distributed	5–30%, >50% in severely infested fields
Insect pests, weevil	*Cylas formicarius* (Coleoptera: Brentidae)	Adults feed on foliage, vines, and storage root epidermis, forming holes; larvae bore into storage root to form tunnels, causing storage root rot	Short-distance spread via adult flight; long-distance dissemination via infested storage roots	Globally distributed insect pests	20–40%, yield losses under low-input management can be as high as 98%
Insect pests, wireworms	Coleoptera: Elateridae	Bore into sweet potato roots and storage roots, forming irregular tunnels, causing plant death and root rot	Soil-dwelling, dissemination via infested soil	Globally distributed insect pests, especially in northern China	~15% or >30% loss in severely infested fields
Insect pests, aphids	*Myzus persicae*/*Aphis gossypii* (Hemiptera: Aphididae)	Leaf curl, chlorosis and plant weakness	Adult aphids disperse via wind and flight; long-distance dissemination via infested seedlings	Globally distributed insect pests	<5% direct yield loss; indirect yield loss of 20–80% as a virus vector

**Table A2:** Overview of reported mechanisms underlying sweet potato responses to biotic stress.

Gene/Protein Name	Types of Biotic Stress	Mechanism	References
*IbMIPS1*	Stem nematodes	Overexpression of *IbMIPS1* in sweet potato plants led to significantly increased contents of inositol, IP_3_, PA, Ca^2+^, ABA, callose and lignin, and markedly enhanced resistance to stem nematodes.	[21]
*IbPIF1* and *IbMVD*	Stem nematodes	IbPIF1 binds to the promoter of *IbMVD* and up-regulates its expression level, thereby promoting terpenoid biosynthesis. Furthermore, up-regulation of *IbPIF1* expression increases the levels of JA, ROS, lignin and callose. These factors are beneficial for enhancing sweet potato resistance to stem nematode.	[67]
*IbBBX24*, *IbJAZ10* and *IbMYC2*	*Fusarium* wilt	In *OE-IbBBX24* plants, IbBBX24 binds to the promoters of *IbJAZ10* and *IbMYC2*, respectively, and regulates their expression levels. Furthermore, IbBBX24 competes with IbJAZ10 for binding to IbMYC2, the released IbMYC2 promotes the expression of downstream JA-response genes, thereby enhancing sweet potato resistance to *Fusarium* wilt.	[137]
*IbCHYR1* and *IbZnFR*	*Fusarium* wilt and *Fusarium* root rot	*Fusarium* effector FfRlpA2 stabilizes and hijacks IbCHYR1 to promote IbZnFR degradation, which in turn abolishes the activation of downstream defense genes by IbZnFR and facilitates *Fusarium* infection.	[42]
*IbNF-YA3*	*Fusarium* root rot	The effector FfRP752 interacts with IbNF-YA3 and promotes its degradation, resulting in the failure of downstream SA-responsive genes to be activated, thereby facilitating pathogen infection.	[138]
*IbJAZ10* and *IbNF-YA3*	*Rhizopus* soft rot	RsSUN41 hijacks and stabilizes IbJAZ10 to promote the degradation of IbNF-YA3, resulting in the failure of downstream *IbPDF1.2*, *IbLOX3*, *IbCALS11* and *IbAP1* to be activated, thereby facilitating pathogen infection in sweet potato.	[139]
*IbPIRL8 and IbDEAR2*	*Fusarium* root rot *Rhizopus* soft rot	IbDEAR2 directly regulates *IbPIRL8* transcription by binding to its promoter, and the resulting IbPIRL8 protein subsequently activates multiple defense pathways, leading to enhanced resistance against both soft rot and root rot in sweet potato.	[144]
*IbSWEET10*	*Fusarium oxysporum*	*IbSWEET10* encodes a sucrose transporter and is strongly upregulated in sweet potato infected by *Fusarium oxysporum*. Overexpression of *IbSWEET10* improved resistance to this pathogen, whereas RNAi lines showed increased susceptibility.	[22]
*IbSPWR1* and *IbSPWR2*	Sweet potato weevil	SPW infestation induces the expression of *IbSPWR1*, which binds to the W-box elements of *IbSPWR2* and promotes its transcription. IbSPWR2 promotes the accumulation of quinate biosynthesis that confers resistance against SPW in sweet potato.	[78]
*IbPAL*, *IbC4H*, and *IbHQT*	Sweet potato weevil	SPW infestation induces the accumulation of the phytohormones JA, SA and ABA. These phytohormones upregulate the expression levels of chlorogenic acid biosynthesis-related genes including *IbPAL*, *IbC4H* and *IbHQT*, thereby promoting chlorogenic acid production. Chlorogenic acid reduces SPW infestation and thus protects sweet potato from damage.	[131]
*Cry1Aa*	*Spodoptera litura*	Overexpression of the *Cry1Aa* gene in sweet potato significantly inhibited the growth of larvae of *Spodoptera litura*, reduced larval body weight and increased larval mortality.	[201]
*IbSPF1* and *IbMPK3/6*	Bacterial pathogen	Specific physical interactions were identified between IbSPF1 and IbMPK3/6, which are capable of phosphorylating the Ser75 and Ser110 residues of IbSPF1.	[161]
*IbNAC1*	Wounding	IbbHLH3 homodimers activate *IbNAC1* in early wounding to trigger defense, while IbbHLH4 forms inhibitory heterodimers with IbbHLH3 in late wounding to terminate the response, with JAZs and IbEIL1 repressing IbbHLH3 under normal conditions to balance growth and defense.	[202]
Coat protein	SPFMV	Introduction of the CP coding gene into sweet potato confers enhanced resistance to SPFMV.	[141]
*miR397* and *IbLAC*	SPVD	Suppression of *miR397* expression in sweet potato via the STTM technique up-regulates the expression of *IbLACs*, activates lignin biosynthesis-related genes, and enhances resistance against SPVD in transgenic plants.	[143]
SPCSV-RNase3	SPVD	Transgenic sweet potato plants harboring the RNase3-targeted RfxCas13d system displayed significantly enhanced resistance to SPVD.	[64]

**Table A3:** Key questions to be addressed in future research based on the reported mechanisms of biotic stress resistance.

	Key Questions
1	Are *IbBBX24* and *IbPIF1* also involved in regulating sweet potato resistance to stem nematodes and *Fusarium* wilt, respectively, and is the former directly regulated by *IbPIF1*?
2	Pathogen effector proteins hijack and regulate host protein stability, thereby facilitating pathogen invasion. Is this a conserved mechanism?
3	Does manipulation of previously characterized biotic stress resistance genes (e.g., *IbBBX24, IbPIF1, IbJAZ10, IbNF-YA3* and *IbPIRL8*) confer broad-spectrum resistance in sweet potato (*Ipomoea batatas*)?
4	How does sweet potato defend against infection by viruses such as SPFMV and SPCSV?
5	What are the core genes co-regulated under both biotic and abiotic stresses in sweet potato, and what are their regulatory networks?
6	How can the reported biotic stress-related genes be utilized in sweet potato molecular breeding?
7	How can emerging technologies (e.g., AI) be employed to accelerate the cloning of biotic stress-related genes and breeding in sweet potato?

**Table A4:** Future strategies for biotic stress management.

	**Perspective**
1	Establish an early warning system for sweet potato pests and diseases, and strengthen international and regional cooperation to facilitate information sharing.
2	Adopt rational cultivation practices and emphasize the modulation of soil microbial communities.
3	Reduce the production and planting costs of virus-free sweet potato seedlings.
4	Strengthen the collection, evaluation, utilization and long-term conservation of sweet potato germplasm resources.
5	Promote the application of emerging biotechnologies (e.g., AI, gene editing, synthetic biology, single-cell and spatial transcriptomics) in sweet potato breeding and mechanism research.
6	Strengthen the integrated application of multi-omics technologies to elucidate the molecular regulatory networks underlying biotic stress responses in sweet potato.
7	Further strengthen the cloning and functional analysis of biotic stress-related genes to elucidate the regulatory networks underlying biotic stress resistance in sweet potato.

## References

[1] Wheeler T., von Braun J. (2013). Climate change impacts on global food security. Science.

[2] Gao L., Cui X. (2023). Climate change and food security: Plant science roles. Mol. Plant.

[3] Sarker P.K., Paul A.S., Karmoker D. (2023). Mitigating climate change and pandemic impacts on global food security: Dual sustainable agriculture approach (2S approach). Planta.

[4] Coast O., Posch B.C., Rognoni B.G., Bramley H., Gaju O., Mackenzie J., Pickles C., Kelly A.M., Lu M., Ruan Y.L. (2022). Wheat photosystem II heat tolerance: Evidence for genotype-by-environment interactions. Plant J..

[5] Mao H., Li S., Chen B., Jian C., Mei F., Zhang Y., Li F., Chen N., Li T., Du L. (2022). Variation in *cis*-regulation of a NAC transcription factor contributes to drought tolerance in wheat. Mol. Plant.

[6] Chang Y., Fang Y., Liu J., Ye T., Li X., Tu H., Ye Y., Wang Y., Xiong L. (2024). Stress-induced nuclear translocation of ONAC023 improves drought and heat tolerance through multiple processes in rice. Nat. Commun..

[7] Guo S., Chen Y., Ju Y., Pan C., Shan J., Ye W., Dong N., Kan Y., Yang Y., Zhao H. (2025). Fine-tuning gibberellin improves rice alkali-thermal tolerance and yield. Nature.

[8] Xiang Y., Liu W., Niu Y., Li Q., Zhao C., Pan Y., Li G., Bian X., Miao Y., Zhang A. (2025). The maize GSK3-like kinase ZmSK1 negatively regulates drought tolerance by phosphorylating the transcription factor ZmCPP2. Plant Cell.

[9] Csikós N., Tóth G. (2023). Concepts of agricultural marginal lands and their utilisation: A review. Agric. Syst..

[10] Sugumar T., Shen G., Smith J., Zhang H. (2024). Creating Climate-Resilient Crops by Increasing Drought, Heat, and Salt Tolerance. Plants.

[11] Akomeah B., Quain M.D., Ramesh S.A., Anand L., López C.M.R. (2019). Common garden experiment reveals altered nutritional values and DNA methylation profiles in micropropagated three elite Ghanaian sweet potato genotypes. PLoS ONE.

[12] Ali A., Bhattacharjee B. (2023). Nutrition security, constraints, and agro-diversification strategies of neglected and underutilized crops to fight global hidden hunger. Front. Nutr..

[13] El Sheikha A.F., Ray R.C. (2017). Potential impacts of bioprocessing of sweet potato: Review. Crit. Rev. Food Sci..

[14] Friedmann M., Asfaw A., Anglin N.L., Becerra L.A., Bhattacharjee R., Brown A., Carey E., Ferguson M.E., Gemenet D., Lindqvist-Kreuze H. (2018). Genomics-Assisted Breeding in the CGIAR Research Program on Roots, Tubers and Bananas (RTB). Agriculture.

[15] Price E.J., Drapal M., Perez-Fons L., Amah D., Bhattacharjee R., Heider B., Rouard M., Swennen R., Becerra Lopez-Lavalle L.A., Fraser P.D. (2020). Metabolite database for root, tuber, and banana crops to facilitate modern breeding in understudied crops. Plant J..

[16] Sapakhova Z., Raissova N., Daurov D., Zhapar K., Daurova A., Zhigailov A., Zhambakin K., Shamekova M. (2023). Sweet Potato as a Key Crop for Food Security under the Conditions of Global Climate Change: A Review. Plants.

[17] Zhang P., Chen C., Shen Y., Ding T., Ma D., Hua Z., Sun D. (2013). Starch saccharification and fermentation of uncooked sweet potato roots for fuel ethanol production. Bioresour. Technol..

[18] Huang Y., Jin Y., Shen W., Fang Y., Zhang G., Zhao H. (2014). The use of plant cell wall-degrading enzymes from newly isolated *Penicillium ochrochloron* Biourge for viscosity reduction in ethanol production with fresh sweet potato tubers as feedstock. Biotechnol. Appl. Biochem..

[19] Lyu R., Ahmed S., Fan W., Yang J., Wu X., Zhou W., Zhang P., Yuan L., Wang H. (2021). Engineering Properties of Sweet Potato Starch for Industrial Applications by Biotechnological Techniques including Genome Editing. Int. J. Mol. Sci..

[20] Yooyongwech S., Samphumphuang T., Tisarum R., Theerawitaya C., Cha-um S. (2016). Arbuscular mycorrhizal fungi (AMF) improved water deficit tolerance in two different sweet potato genotypes involves osmotic adjustments via soluble sugar and free proline. Sci. Hortic..

[21] Zhai H., Wang F., Si Z., Huo J., Xing L., An Y., He S., Liu Q. (2016). A *myo*-inositol-1-phosphate synthase gene, *IbMIPS1*, enhances salt and drought tolerance and stem nematode resistance in transgenic sweet potato. Plant Biotechnol. J..

[22] Li Y., Wang Y., Zhang H., Zhang Q., Zhai H., Liu Q., He S. (2017). The Plasma Membrane-Localized Sucrose Transporter IbSWEET10 Contributes to the Resistance of Sweet Potato to *Fusarium oxysporum*. Front. Plant Sci..

[23] Peck S., Mittler R. (2020). Plant signaling in biotic and abiotic stress. J. Exp. Bot..

[24] Li X., Liu M., Huang T., Yang K., Zhou S., Li Y., Tian J. (2021). Antifungal effect of nerol via transcriptome analysis and cell growth repression in sweet potato spoilage fungi *Ceratocystis fimbriata*. Postharvest Biol. Technol..

[25] Rutter W.B., Wadl P.A., Mueller J.D., Agudelo P. (2021). Identification of Sweet Potato Germplasm Resistant to Pathotypically Distinct Isolates of *Meloidogyne enterolobii* from the Carolinas. Plant Dis..

[26] Yang Y., Chen Y., Bo Y., Liu Q., Zhai H. (2023). Research Progress in the Mechanisms of Resistance to Biotic Stress in Sweet Potato. Genes.

[27] Song B., Raza A., He F., Wang S., Huang X., Mo A., Jiang K., Guo J., Srivastava A.K., Riaz A. (2025). Recent advances in miRNA and siRNA approaches, and genome editing to augment biotic and abiotic stress tolerance in sweet potato (*Ipomoea batatas* L.). Int. J. Biol. Macromol..

[28] Rouphael Y., Kyriacou M.C., Colla G. (2018). Vegetable Grafting: A Toolbox for Securing Yield Stability under Multiple Stress Conditions. Front. Plant Sci..

[29] Wang J., Chen P., Zhao T., Huang X., Zong J., Luo Q., Peng C., Wu X., Qiu F., Zhao D. (2024). Biosynthesis of Scopoletin in Sweet Potato Confers Resistance against *Fusarium oxysporum*. J. Agric. Food Chem..

[30] Adero J., Akongo G.O., Yada B., Byarugaba D.K., Kitavi M., Bua B., Yencho G.C., Otema M.A. (2024). Sweet Potato Virus Disease and Its Associated Vectors: Farmers’ Knowledge and Management Practices in Uganda. J. Agric. Sci..

[31] Morales A., Ma P., Jia Z., Rodríguez D., Vargas I.J.P., González R.E., Molina O., Jiménez A., Rodríguez Y., Morales L. (2025). Decoding phenotypic signatures of *Cylas formicarius* Fab. resistance in a global sweetpotato (*Ipomoea batatas* [L.] Lam.) germplasm collection. Front. Plant Sci..

[32] Gurr G.M., Liu J., Johnson A.C., Woruba D.N., Kirchhof G., Fujinuma R., Sirabis W., Jeffery Y., Akkinapally R. (2016). Pests, diseases and crop protection practices in the smallholder sweetpotato production system of the highlands of Papua New Guinea. PeerJ.

[33] Lister A.M. (2009). The biotic effects of climate change. Clin. Med..

[34] Teshome D.T., Zharare G.E., Naidoo S. (2020). The Threat of the Combined Effect of Biotic and Abiotic Stress Factors in Forestry Under a Changing Climate. Front. Plant Sci..

[35] Zandalinas S.I., Fritschi F.B., Mittler R. (2021). Global Warming, Climate Change, and Environmental Pollution: Recipe for a Multifactorial Stress Combination Disaster. Trends Plant Sci..

[36] Choudhary R., Ahmad F., Kaya C., Upadhyay S.K., Muneer S., Kumar V., Meena M., Liu H., Upadhyaya H., Seth C.S. (2025). Decrypting proteomics, transcriptomics, genomics, and integrated omics for augmenting the abiotic, biotic, and climate change stress resilience in plants. J. Plant Physiol..

[37] Xing K., Li T., Liu Y., Zhang J., Zhang Y., Shen X., Li X., Miao X., Feng Z., Peng X. (2018). Antifungal and eliciting properties of chitosan against *Ceratocystis fimbriata* in sweet potato. Food Chem..

[38] Gao Z., Han M., Hu Y., Li Z., Liu C., Wang X., Tian Q., Jiao W., Hu J., Liu L. (2019). Effects of Continuous Cropping of Sweet Potato on the Fungal Community Structure in *Rhizospheric* Soil. Front. Microbiol..

[39] Cheng J., Wu J., Liu Z., Zhang X., Lu X., Yin L., Lu G., Pang L. (2023). Identifying Early-Stage Changes in Volatile Organic Compounds of *Ceratocystis fimbriata* Ellis & Halsted-Infected Sweet Potatoes (*Ipomoea batatas* L. Lam) Using Headspace Gas Chromatography-Ion Mobility Spectrometry. Foods.

[40] Cong H., Li C., Wang Y., Zhang Y., Ma D., Li L., Jiang J. (2023). The Mechanism of Transcription Factor Swi6 in Regulating Growth and Pathogenicity of *Ceratocystis fimbriata*: Insights from Non-Targeted Metabolomics. Microorganisms.

[41] Lee J., Ji J., Ryu S., Park J.W., Paul N.C., Park S., Im S., Han G., Sang H. (2025). Control of sweet potato *Fusarium* wilt and black rot by endophytic *Bacillus velezensis* with improved lipopeptide production. BMC Microbiol..

[42] Zhang H., Dai Z., Zhang X., Shang M., Gao X., Ma R., Zhao L., Zhang X., Liu Q., Zhai H. (2025). Natural Allelic Variations in *IbCHYR1-IbZnFR* Complex Regulate *Fusarium* Root Rot Resistance in Sweet Potato. Adv. Sci..

[43] Stahr M.N., Quesada-Ocampo L.M. (2021). Effects of Water Temperature, Inoculum Concentration and Age, and Sanitizers on Infection of *Ceratocystis fimbriata*, Causal Agent of Black Rot in Sweetpotato. Plant Dis..

[44] Kim S., Kim T., Chung M., Lee Y., Lee I., Lee H., Park W. (2022). Incidence Rates of Root Rot in Sweetpotato Caused by Cultivation Soil and Soil Microorganisms During Storage Periods. Front. Plant Sci..

[45] Wang R., Gao B., Li X., Ma J., Chen S. (2014). First Report of *Fusarium solani* Causing *Fusarium* Root Rot and Stem Canker on Storage Roots of Sweet Potato in China. Plant Dis..

[46] Pan C., Yang K., Erhunmwunsee F., Li Y., Liu M., Pan S., Yang D., Lu G., Ma D., Tian J. (2023). Inhibitory effect of cinnamaldehyde on *Fusarium solani* and its application in postharvest preservation of sweet Potato. Food Chem..

[47] Chen S., Kuo Y., Lin J. (2023). Review: Defense responses in sweetpotato (*Ipomoea batatas* L.) against biotic stress. Plant Sci..

[48] Mphela W.M., Laurie S.M., Minnaar-Ontong A., Bihon W. (2022). Development and screening of *Fusarium* wilt resistant lines in Sweet potato [*Ipomoea batatas* (L.) Lam]. Euphytica.

[49] Lu X., Yu S., Yu B., Chen L., Wang Y., Huang Y., Lu G., Cheng J., Guan Y., Yin L. (2024). Biochemical mechanism of chlorine dioxide fumigation in inhibiting *Ceratocystis fimbriata* and black rot in postharvest sweetpotato. Food Chem..

[50] Gutiérrez D.L., Fuentes S., Salazar L.F. (2003). Sweetpotato Virus Disease (SPVD): Distribution, Incidence, and Effect on Sweetpotato Yield in Peru. Plant Dis..

[51] Kreuze J.F., Klein I.S., Lazaro M.U., Chuquiyuri W.J., Morgan G.L., Mejía P.G., Ghislain M., Valkonen J.P. (2008). RNA silencing-mediated resistance to a crinivirus (Closteroviridae) in cultivated sweet potato (*Ipomoea batatas* L.) and development of sweet potato virus disease following co-infection with a potyvirus. Mol. Plant Pathol..

[52] Untiveros M., Fuentes S., Salazar L.F. (2007). Synergistic Interaction of *Sweet potato chlorotic stunt virus (Crinivirus)* with Carla-, Cucumo-, Ipomo-, and Potyviruses Infecting Sweet Potato. Plant Dis..

[53] Bednarek R., David M., Fuentes S., Kreuze J., Fei Z.J. (2021). Transcriptome analysis provides insights into the responses of sweet potato to sweet potato virus disease (SPVD). Virus Res..

[54] Chase O., Javed A., Byrne M.J., Thuenemann E.C., Lomonossoff G.P., Ranson N.A., López-Moya J.J. (2023). CryoEM and stability analysis of virus-like particles of potyvirus and ipomovirus infecting a common host. Commun. Biol..

[55] Villalba A., Martínez-Ispizua E., Morard M., Crespo-Sempere A., Albiach-Marti M.R., Calatayud A., Penella C. (2024). Optimizing sweet potato production: Insights into the interplay of plant sanitation, virus influence, and cooking techniques for enhanced crop quality and food security. Front. Plant Sci..

[56] Adero J., Ssali R., Segundo F., Maria D., Kitavi M., Yada B., Byarugaba D.K., Dube F., Aber P.P., Opiyo S.O. (2025). Virus-Specific Defense Responses in Sweetpotato: Transcriptomic Insights into Resistance and Susceptibility to SPFMV, SPCSV, and SPVD. Biology.

[57] Jo Y., Kim S., Choi H., Yang J., Lee B., Cho W. (2020). Sweet potato viromes in eight different geographical regions in Korea and two different cultivars. Sci. Rep..

[58] Tugume A.K., Mukasa S.B., Valkonen J.P. (2008). Natural Wild Hosts of *Sweet potato feathery mottle virus* Show Spatial Differences in Virus Incidence and Virus-Like Diseases in Uganda. Phytopathology.

[59] Yang X., Li Y., Wang A. (2021). Research Advances in Potyviruses: From the Laboratory Bench to the Field. Annu. Rev. Phytopathol..

[60] Kokkinos C.D., Clark C.A. (2006). Interactions Among *Sweet potato chlorotic stunt virus* and Different Potyviruses and Potyvirus Strains Infecting Sweetpotato in the United States. Plant Dis..

[61] Gamarra H.A., Fuentes S., Morales F.J., Glover R., Malumphy C., Barker I. (2010). *Bemisia afer* sensu lato, a Vector of *Sweet potato chlorotic stunt virus*. Plant Dis..

[62] Zhang K., Lu H., Wan C., Tang D., Zhao Y., Luo K., Li S., Wang J. (2020). The Spread and Transmission of Sweet Potato Virus Disease (SPVD) and Its Effect on the Gene Expression Profile in Sweet Potato. Plants.

[63] Zhang Y., Zhao F., Li J., Li R., Tian T., Qiao Q., Wang Y., Zhang D., Jing X., Wang L. (2025). Transcriptome and small RNA changes in response to coinfection with SPCSV and SPFMV during sweet potato storage root sprouting. Crop Sci..

[64] Yu Y., Pan Z., Wang X., Bian X., Wang W., Liang Q., Kou M., Ji H., Li Y., Ma D. (2022). Targeting of SPCSV-*RNase3* via CRISPR-Cas13 confers resistance against sweet potato virus disease. Mol. Plant Pathol..

[65] Pan F., Li F., Mao Y., Liu D., Chen A., Zhao D., Hu Y. (2021). First Detection of *Ditylenchus destructor* Parasitizing Maize in Northeast China. Life.

[66] Fraher S., Schwarz T., Heim C., De Siqueira Gesteira G., Mollinari M., Da Silva Pereira G., Zeng Z.B., Brown-Guedira G., Gorny A., Yencho G.C. (2024). Discovery of a major QTL for resistance to the guava root-knot nematode (*Meloidogyne enterolobii*) in ‘Tanzania’, an African landrace sweetpotato (*Ipomoea batatas*). Theor. Appl. Genet..

[67] Nie N., Yang Y., Huo J., Wang F., Liu R., Sun S., Hu Y., Chen Y., Wu W., Liu Q. (2025). IbPIF1 confers stem nematode resistance by regulating secondary metabolites in sweet potato. Plant Biotechnol. J..

[68] Jones J.T., Haegeman A., Danchin E.G., Gaur H.S., Helder J., Jones M.G., Kikuchi T., Manzanilla-López R., Palomares-Rius J.E., Wesemael W.M. (2013). Top 10 plant-parasitic nematodes in molecular plant pathology. Mol. Plant Pathol..

[69] Canella Vieira C., Zhou J., Usovsky M., Vuong T., Howland A.D., Lee D., Li Z., Zhou J., Shannon G., Nguyen H.T. (2022). Exploring Machine Learning Algorithms to Unveil Genomic Regions Associated With Resistance to Southern Root-Knot Nematode in Soybeans. Front. Plant Sci..

[70] Alam M.S., Khanal C., Roberts J., Rutter W., Wadl P.A. (2024). Enhancing Reniform Nematode Management in Sweetpotato by Complementing Host-Plant Resistance with Nonfumigant Nematicides. Plant Dis..

[71] Huang L., Fang B., Luo Z., Chen J., Zhang X., Wang Z. (2010). First Report of Bacterial Stem and Root Rot of Sweetpotato Caused by a *Dickeya* sp. (*Erwinia chrysanthemi*) in China. Plant Dis..

[72] Hossain A., Hong X., Ibrahim E., Li B., Sun G., Meng Y., Wang Y., An Q. (2019). Green Synthesis of Silver Nanoparticles with Culture Supernatant of a Bacterium *Pseudomonas rhodesiae* and Their Antibacterial Activity against Soft Rot Pathogen *Dickeya dadantii*. Molecules.

[73] He W., Huang D., Wu J., Li X., Qian Y., Li B., Lou B., Wu J. (2021). Three Highly Sensitive and High-Throughput Serological Approaches for Detecting *Dickeya dadantii* in Sweet Potato. Plant Dis..

[74] Li H., Zhang H., Liu Z., Lin Z., Qiu Y., Tang H., Qiu S. (2021). Rapid diagnosis of *Ralstonia solanacearum* infection sweet potato in China by loop-mediated isothermal amplification. Arch. Microbiol..

[75] Ferreira V., Pianzzola M.J., Vilaró F.L., Galván G.A., Tondo M.L., Rodriguez M.V., Orellano E.G., Valls M., Siri M.I. (2017). Interspecific Potato Breeding Lines Display Differential Colonization Patterns and Induced Defense Responses after *Ralstonia solanacearum* Infection. Front. Plant Sci..

[76] Park Y., Nam H., Baek S., Lee S., Lee J. (2019). Population genetic structure of *Bemisia tabaci* MED (Hemiptera: Aleyrodidae) in Korea. PLoS ONE.

[77] Okada Y., Monden Y., Nokihara K., Shirasawa K., Isobe S., Tahara M. (2019). Genome-Wide Association Studies (GWAS) for Yield and Weevil Resistance in Sweet potato (*Ipomoea batatas* (L.) Lam). Plant Cell Rep..

[78] Liu X., Wang Y., Zhu H., Mei G., Liao Y., Rao S., Li S., Chen A., Liu H., Zeng L. (2022). Natural allelic variation confers high resistance to sweet potato weevils in sweet potato. Nat. Plants.

[79] Chalfant R.B., Jansson R.K., Seal D.R., Schalk J.M. (1990). Ecology and Management of Sweet Potato Insects. Annu. Rev. Entomol..

[80] Arrington A.E., Kennedy G.G., Abney M.R. (2016). Applying insecticides through drip irrigation to reduce wireworm (Coleoptera: Elateridae) feeding damage in sweet potato. Pest. Manag. Sci..

[81] Seal D.R., Baniya A.B., Dyrdahl-Young R., Hochmuth R.C., Leppla N.C., Fenneman D.K., Broughton R.T., DiGennaro P. (2020). Wireworm (Coleoptera: Elateridae) Species Composition and Management in Sweet Potato Grown in North Florida Using Chemical Insecticides and Entomopathogenic Nematodes. Environ. Entomol..

[82] Karyeija R.F., Kreuze J.F., Gibson R.W., Valkonen J.P. (2000). Synergistic Interactions of a Potyvirus and a Phloem-Limited Crinivirus in Sweet Potato Plants. Virology.

[83] Fujita H., Yoshida S., Suzuki K., Toju H. (2024). Soil prokaryotic and fungal biome structures associated with crop disease status across the Japan Archipelago. mSphere.

[84] Ikram A.U., Khan M.S.S., Islam F., Ahmed S., Ling T., Feng F., Sun Z., Chen H., Chen J. (2025). All Roads Lead to Rome: Pathways to Engineering Disease Resistance in Plants. Adv. Sci..

[85] Karyeija R.F., Gibson R.W., Valkonen J.P.T. (1998). The Significance of Sweet Potato Feathery Mottle Virus in Subsistence Sweet Potato Production in Africa. Plant Dis..

[86] Dastmalchi K., Perez Rodriguez M., Lin J., Yoo B., Stark R.E. (2019). Temporal resistance of potato tubers: Antibacterial assays and metabolite profiling of wound-healing tissue extracts from contrasting cultivars. Phytochemistry.

[87] Veronico P., Sasanelli N., Troccoli A., Myrta A., Midthassel A., Butt T. (2023). Evaluation of Fungal Volatile Organic Compounds for Control the Plant Parasitic Nematode *Meloidogyne incognita*. Plants.

[88] Jones J.D.G., Staskawicz B.J., Dangl J.L. (2024). The plant immune system: From discovery to deployment. Cell.

[89] Okada Y., Kobayashi A., Tabuchi H., Kuranouchi T. (2017). Review of major sweetpotato pests in Japan, with information on resistance breeding programs. Breed. Sci..

[90] Wang Y., Qin Y., Wang S., Zhang D., Tian Y., Zhao F., Wang Y., Lv H., Qiao Q., Zhang Z. (2021). Species and genetic variability of sweet potato viruses in China. Phytopathol. Res..

[91] Gibson R.W., Aritua V., Byamukama E., Mpembe I., Kayongo J. (2004). Control strategies for sweet potato virus disease in Africa. Virus Res..

[92] Mulabisana M.J., Cloete M., Mabasa S.M., Laurie S.M., Oelofse D., Esterhuizen L.L., Rey M.E.C. (2018). Surveys in the Gauteng, Limpopo and Mpumalanga provinces of South Africa reveal novel isolates of sweet potato viruses. S. Afr. J. Bot..

[93] Ngailo S., Shimelis H., Sibiya J., Mtunda K., Mashilo J. (2019). Genotype-by-environment interaction of newly-developed sweet potato genotypes for storage root yield, yield-related traits and resistance to sweet potato virus disease. Heliyon.

[94] Okonya J.S., Ocimati W., Nduwayezu A., Kantungeko D., Niko N., Blomme G., Legg J.P., Krosche J. (2019). Farmer Reported Pest and Disease Impacts on Root, Tuber, and Banana Crops and Livelihoods in Rwanda and Burundi. Sustainability.

[95] Kreuze J.F., Perez A., Gargurevich M.G., Cuellar W.J. (2020). Badnaviruses of Sweet Potato: Symptomless Coinhabitants on a Global Scale. Front. Plant Sci..

[96] Ogero K., Okuku H.S., Wanjala B., McEwan M., Almekinders C., Kreuze J., Struik P., van der Vlugt R. (2023). Degeneration of cleaned-up, virus-tested sweetpotato seed vines in Tanzania. Crop Prot..

[97] Laurie S.M., Mulabisana J., Sutherland R., Sivakumar D., Pofu K., Mphela W.M., Truter M., Plooy I.D., Araya N., Araya H. (2023). Seventy years of sweet potato [*Ipomoea batatas* L. (LAM)] research in South Africa. Crop Sci..

[98] Cabral M.J.S., Haseeb M., Soares M.A. (2024). Major Insect Pests of Sweet Potatoes in Brazil and the United States, with Information on Crop Production and Regulatory Pest Management. Insects.

[99] Maeda A., Minoshima A., Kawano S., Nakamura M., Takushi T., Yamashiro M., Kawamura F., Oshiro A., Ichinose K., Okada Y. (2022). Foot rot disease of sweet potato in Japan caused by *Diaporthe destruens*: First report, pathogenicity and taxonomy. J. Gen. Plant Pathol..

[100] Byamukama E., Gibson R.W., Aritua V., Adipala E. (2004). Within-crop spread of sweet potato virus disease and the population dynamics of its whitefly and aphid vectors. Crop Prot..

[101] Valverde R.A., Sim J., Lotrakul P. (2004). Whitefly transmission of sweet potato viruses. Virus Res..

[102] Abad J.A., Parks E.J., New S.L., Fuentes S., Jester W., Moyer J.W. (2007). First Report of *Sweet potato chlorotic stunt virus*, a Component of Sweetpotato Virus Disease, in North Carolina. Plant Dis..

[103] Luan Y., Zhang J., An L. (2006). First Report of *Sweet potato leaf curl virus* in China. Plant Dis..

[104] Gao B., Wang R., Chen S., Li X., Ma J. (2014). First Report of Root-Knot Nematode *Meloidogyne enterolobii* on Sweet Potato in China. Plant Dis..

[105] Huang L., Zhang X., Yang Y., Zou H., Fang B., Liu W. (2021). High-Quality Genome Resource of *Diaporthe destruens* Causing Foot Rot Disease of Sweet Potato. Plant Dis..

[106] Addo-Bediako A., Tameru B., Jackai L.E., Bonsi C.K. (2007). Assessment of risk of introduction of *Cylas formicarius elegantulus* (Coleoptera: Brentidae) into weevil-free areas in the southern United States. J. Econ. Entomol..

[107] Okonya J., Okonya J.S., Kroschel J. (2013). Incidence, abundance and damage by the sweet potato butterfly (*Acraea acerata* Hew. and the African sweet potato weevils (*Cylas* spp.) across an altitude gradient in Kabale district, Uganda. Int. J. Agric. Sci..

[108] Wu S., Sun H., Zhao X., Hamilton J.P., Mollinari M., Gesteira G.S., Kitavi M., Yan M., Wang H., Yang J. (2025). Phased chromosome-level assembly provides insight into the genome architecture of hexaploid sweetpotato. Nat. Plants.

[109] Hirakawa H., Okada Y., Tabuchi H., Shirasawa K., Watanabe A., Tsuruoka H., Minami C., Nakayama S., Sasamoto S., Kohara M. (2015). Survey of genome sequences in a wild sweet potato, *Ipomoea trifida* (H. B. K.) G. Don. DNA Res..

[110] Wu S., Lau K.H., Cao Q., Hamilton J.P., Sun H., Zhou C., Eserman L., Gemenet D.C., Olukolu B.A., Wang H. (2018). Genome sequences of two diploid wild relatives of cultivated sweetpotato reveal targets for genetic improvement. Nat. Commun..

[111] Yang J., Moeinzadeh M.H., Kuhl H., Helmuth J., Xiao P., Haas S., Liu G., Zheng J., Sun Z., Fan W. (2017). Haplotype-resolved sweet potato genome traces back its hexaploidization history. Nat. Plants.

[112] Yan M., Li M., Wang Y., Wang X., Moeinzadeh M.H., Quispe-Huamanquispe D.G., Fan W., Fang Y., Wang Y., Nie H. (2024). Haplotype-based phylogenetic analysis and population genomics uncover the origin and domestication of sweetpotato. Mol. Plant.

[113] López-Márquez D., Del-Espino Á., López-Pagán N., Rodríguez-Negrete E.A., Rubio-Somoza I., Ruiz-Albert J., Bejarano E.R., Beuzón C.R. (2021). miR825-5p targets the TIR-NBS-LRR gene *MIST1* and down-regulates basal immunity against *Pseudomonas syringae* in Arabidopsis. J. Exp. Bot..

[114] Schulze S., Yu L., Hua C., Zhang L., Kolb D., Weber H., Ehinger A., Saile S.C., Stahl M., Franz-Wachtel M. (2022). The *Arabidopsis* TIR-NBS-LRR protein CSA1 guards BAK1-BIR3 homeostasis and mediates convergence of pattern- and effector-induced immune responses. Cell Host Microbe.

[115] Sett S., Prasad A., Prasad M. (2022). Resistance genes on the verge of plant-virus interaction. Trends Plant Sci..

[116] Xiao S., Wang Y., Zhou Z., Zhao L., Zhao L., Gao B., Dai X., Xu P., Cao Q. (2024). Xiaoshu, a simple genetic model system for sweetpotato (*Ipomoea batatas* (L.) Lam.). Plant Biotechnol. J..

[117] Ahmed S., Khan M.S.S., Xue S., Islam F., Ikram A.U., Abdullah M., Liu S., Tappiban P., Chen J. (2024). A comprehensive overview of omics-based approaches to enhance biotic and abiotic stress tolerance in sweet potato. Hortic. Res..

[118] Tao X., Gu Y., Wang H., Zheng W., Li X., Zhao C., Zhang Y. (2012). Digital Gene Expression Analysis Based on Integrated *De Novo* Transcriptome Assembly of Sweet Potato [*Ipomoea batatas* (L.) Lam]. PLoS ONE.

[119] Lin Y., Zou W., Lin S., Onofua D., Yang Z., Chen H., Wang S., Chen X. (2017). Transcriptome profiling and digital gene expression analysis of sweet potato for the identification of putative genes involved in the defense response against *Fusarium oxysporum* f. sp. *batatas*. PLoS ONE.

[120] Leivar P., Monte E. (2014). PIFs: Systems Integrators in Plant Development. Plant Cell.

[121] Zhang L., He C., Lai Y., Wang Y., Kang L., Liu A., Lan C., Su H., Gao Y., Li Z. (2023). Asymmetric gene expression and cell-type-specific regulatory networks in the root of bread wheat revealed by single-cell multiomics analysis. Genome Biol..

[122] Yuan Y., Huo Q., Zhang Z., Wang Q., Wang J., Chang S., Cai P., Song K., Galbraith D.W., Zhang W. (2024). Decoding the gene regulatory network of endosperm differentiation in maize. Nat. Commun..

[123] Yao J., Chu Q., Guo X., Shao W., Shang N., Luo K., Li X., Chen H., Cheng Q., Mo F. (2024). Spatiotemporal transcriptomic landscape of rice embryonic cells during seed germination. Dev. Cell.

[124] Wang T., Wang F., Deng S., Wang K., Feng D., Xu F., Guo W., Yu J., Wu Y., Wuriyanghan H. (2025). Single-cell transcriptomes reveal spatiotemporal heat stress response in maize roots. Nat. Commun..

[125] Zhu M., Hsu C.W., Peralta Ogorek L.L., Taylor I.W., La Cavera S., Oliveira D.M., Verma L., Mehra P., Mijar M., Sadanandom A. (2025). Single-cell transcriptomics reveal how root tissues adapt to soil stress. Nature.

[126] Zhang M., Li W., Zhang T., Liu Y., Liu L. (2024). *Botrytis cinerea*-induced F-box protein 1 enhances disease resistance by inhibiting JAO/JOX-mediated jasmonic acid catabolism in *Arabidopsis*. Mol. Plant.

[127] Huang S., Wang C., Ding Z., Zhao Y., Dai J., Li J., Huang H., Wang T., Zhu M., Feng M. (2024). A plant NLR receptor employs ABA central regulator PP2C-SnRK2 to activate antiviral immunity. Nat. Commun..

[128] Spoel S., Dong X. (2024). Salicylic acid in plant immunity and beyond. Plant Cell.

[129] Jia H., Hewitt N., Jordá L., Abramyan T.M., Tolliver J., Jones J.L., Nomura K., Yang J., He S.Y., Tropsha A. (2025). Phosphorylation-activated G protein signaling stabilizes TCP14 and JAZ3 to repress JA signaling and enhance plant immunity. Mol. Plant.

[130] Tian H., Xu L., Li X., Zhang Y. (2025). Salicylic acid: The roles in plant immunity and crosstalk with other hormones. J. Integr. Plant Biol..

[131] Liao Y., Zeng L., Rao S., Gu D., Liu X., Wang Y., Zhu H., Hou X., Yang Z. (2020). Induced biosynthesis of chlorogenic acid in sweetpotato leaves confers the resistance against sweetpotato weevil attack. J. Adv. Res..

[132] Nokihara K., Okada Y., Ohata S., Monden Y. (2021). Transcriptome Analysis Reveals Key Genes Involved in Weevil Resistance in the Hexaploid Sweetpotato. Plants.

[133] Huang X., Xu C., Yang S., Li J., Wang H., Zhang Z., Chen C., Xie H. (2019). Life-stage specific transcriptomes of a migratory endoparasitic plant nematode, *Radopholus similis* elucidate a different parasitic and life strategy of plant parasitic nematodes. Sci. Rep..

[134] Mathew R., Opperman C.H. (2020). Current Insights into Migratory Endoparasitism: Deciphering the Biology, Parasitism Mechanisms, and Management Strategies of Key Migratory Endoparasitic Phytonematodes. Plants.

[135] Nie N., Huo J., Sun S., Zuo Z., Chen Y., Liu Q., He S., Gao S., Zhang H., Zhao N. (2023). Genome-Wide Characterization of the PIFs Family in Sweet Potato and Functional Identification of *IbPIF3.1* under Drought and *Fusarium* Wilt Stresses. Int. J. Mol. Sci..

[136] Nagegowda D.A., Gupta P. (2020). Advances in biosynthesis, regulation, and metabolic engineering of plant specialized terpenoids. Plant Sci..

[137] Zhang H., Zhang Q., Zhai H., Gao S., Yang L., Wang Z., Xu Y., Huo J., Ren Z., Zhao N. (2020). IbBBX24 Promotes the Jasmonic Acid Pathway and Enhances *Fusarium* Wilt Resistance in Sweet Potato. Plant Cell.

[138] Shang M., Zhang X., Zhang X., Yan J., Liu Q., Zhai H., Gao S., Zhao N., He S., Zhang H. (2025). A chromosome-level genome assembly of *Fusarium foetens* that causes sweet potato root rot facilitates the identification of a key *Fusarium-specific pathogenicity* factor. Plant Commun..

[139] Wang W., Wang Z., Wei Y., Shang M., Ma R., Zhao L., Zhai H., Gao S., Zhao N., Liu Q. (2026). Natural allelic variations in IbJAZ10-IbNF-YA3 complex regulate *Rhizopus* soft rot resistance in sweet potato. N. Phytol..

[140] Kreuze J.F., Ramírez D.A., Fuentes S., Loayza H., Ninanya J., Rinza J., David M., Gamboa S., De Boeck B., Diaz F. (2024). High-throughput characterization and phenotyping of resistance and tolerance to virus infection in sweetpotato. Virus Res..

[141] Okada Y., Saito A., Nishiguchi M., Kimura T., Mori M., Hanada K., Sakai J., Miyazaki C., Matsuda Y., Murata T. (2001). Virus resistance in transgenic sweetpotato [*Ipomoea batatas* L. (Lam)] expressing the coat protein gene of sweet potato feathery mottle virus. Theor. Appl. Genet..

[142] Sivparsad B.J., Gubba A. (2014). Development of transgenic sweet potato with multiple virus resistance in South Africa (SA). Transgenic Res..

[143] Li C., Liu X., Abouelnasr H., Mohamed H., Kou M., Tang W., Yan H., Wang X., Wang X., Zhang Y. (2022). Inhibition of miR397 by STTM technology to increase sweetpotato resistance to SPVD. J. Integr. Agric..

[144] Wang W., Peng K., Shang M., Zhai H., Gao S., Zhao N., Liu Q., Zhang H., He S. (2026). An intracellular Ras-group related leucine-rich repeat protein IbPIRL8 increases soft rot and root rot resistance in sweet potato. Crop J..

[145] Block A.K., Vaughan M.M., Schmelz E.A., Christensen S.A. (2019). Biosynthesis and function of terpenoid defense compounds in maize (*Zea mays*). Planta.

[146] Cagnola J.I., Cerdán P.D., Pacín M., Andrade A., Rodriguez V., Zurbriggen M.D., Legris M., Buchovsky S., Carrillo N., Chory J. (2018). Long-Day Photoperiod Enhances Jasmonic Acid-Related Plant Defense. Plant Physiol..

[147] Yu X., Niu H., Liu C., Wang H., Yin W., Xia X. (2024). PTI-ETI synergistic signal mechanisms in plant immunity. Plant Biotechnol. J..

[148] Suzuki N., Rivero R.M., Shulaev V., Blumwald E., Mittler R. (2014). Abiotic and biotic stress combinations. N. Phytol..

[149] Saijo Y., Loo E.P.I. (2020). Plant immunity in signal integration between biotic and abiotic stress responses. N. Phytol..

[150] Liu J., Su L., Wang W., Wang J., Chen W., Yang Z., Yang L., Li G., Liu X., Zhang H. (2025). Light-induced OsLIKE1 phosphorylation enhances rice resistance against blast disease. Nat. Commun..

[151] Letsch H., Gottsberger B., Metzl C., Astrin J., Friedman A.L.L., McKenna D.D., Fiedler K. (2018). Climate and host-plant associations shaped the evolution of ceutorhynch weevils throughout the Cenozoic. Evolution.

[152] Xue Y., Lin C., Wang Y., Liu W., Wan F., Zhang Y., Ji L. (2022). Predicting Climate Change Effects on the Potential Distribution of Two Invasive Cryptic Species of the Bemisia tabaci Species Complex in China. Insects.

[153] Karuri H., Olago D., Neilson R., Njeri E., Opere A., Ndegwa P. (2017). Plant parasitic nematode assemblages associated with sweet potato in Kenya and their relationship with environmental variables. Trop. Plant Pathol..

[154] Bohatá A., Folorunso E.A., Lencová J., Osborne L.S., Mraz J. (2024). Control of sweet potato whitefly (*Bemisia tabaci*) using entomopathogenic fungi under optimal and suboptimal relative humidity conditions. Pest. Manag. Sci..

[155] Pandey P., Senthil-Kumar M. (2019). Plant-pathogen interaction in the presence of abiotic stress: What do we know about plant responses?. Plant Physiol. Rep..

[156] Zarattini M., Farjad M., Launay A., Cannella D., Soulié M.C., Bernacchia G., Fagard M. (2021). Every cloud has a silver lining: How abiotic stresses affect gene expression in plant-pathogen interactions. J. Exp. Bot..

[157] Meng X., Zhang S. (2013). MAPK Cascades in Plant Disease Resistance Signaling. Annu. Rev. Phytopathol..

[158] Bigeard J., Colcombet J., Hirt H. (2015). Signaling Mechanisms in Pattern-Triggered Immunity (PTI). Mol. Plant.

[159] Sun T.J., Zhang Y.L. (2022). MAP kinase cascades in plant development and immune signaling. EMBO Rep..

[160] Kim H.S., Park S.C., Ji C.Y., Park S., Jeong J.C., Lee H.S., Kwak S.S. (2016). Molecular characterization of biotic and abiotic stress-responsive MAP kinase genes, *IbMPK3* and *IbMPK6*, in sweetpotato. Plant Physiol. Biochem..

[161] Kim H., Bian X., Lee C., Kim S., Park S., Xie Y., Guo X., Kwak S. (2019). IbMPK3/IbMPK6-mediated IbSPF1 phosphorylation promotes tolerance to bacterial pathogen in sweetpotato. Plant Cell Rep..

[162] Li S., Feng Z., Yang B., Li H., Liao F., Gao Y., Liu S., Tang J., Yao Q. (2022). An intelligent monitoring system of diseases and pests on rice canopy. Front. Plant Sci..

[163] Liu C., Zhai Z., Zhang R., Bai J., Zhang M. (2022). Field pest monitoring and forecasting system for pest control. Front. Plant Sci..

[164] Gao Z., Hu Y., Han M., Xu J., Wang X., Liu L., Tang Z., Jiao W., Jin R., Liu M. (2021). Effects of continuous cropping of sweet potatoes on the bacterial community structure in rhizospheric soil. BMC Microbiol..

[165] Johnson A.W., Dowler C.C., Glaze N.C., Handoo Z.A. (1996). Role of Nematodes, Nematicides, and Crop Rotation on the Productivity and Quality of Potato, Sweet Potato, Peanut, and Grain Sorghum. J. Nematol..

[166] Larkin R.P., Griffin T.S., Honeycutt C.W. (2010). Rotation and Cover Crop Effects on Soilborne Potato Diseases, Tuber Yield, and Soil Microbial Communities. Plant Dis..

[167] Xie W., Zhu A., Ali T., Zhang Z., Chen X., Wu F., Huang J., Davis K.F. (2023). Crop switching can enhance environmental sustainability and farmer incomes in China. Nature.

[168] Yang X., Xiong J., Du T., Ju X., Gan Y., Li S., Xia L., Shen Y., Pacenka S., Steenhuis T.S. (2024). Diversifying crop rotation increases food production, reduces net greenhouse gas emissions and improves soil health. Nat. Commun..

[169] Andreason S.A., McKenzie-Reynolds P., Whitley K.M., Coffey J., Simmons A.M., Wadl P.A. (2024). Tracking Sweet Potato Leaf Curl Virus through Field Production: Implications for Sustainable Sweetpotato Production and Breeding Practices. Plants.

[170] Li H., Wang J., Liu Q., Zhou Z., Chen F., Xiang D. (2019). Effects of consecutive monoculture of sweet potato on soil bacterial community as determined by pyrosequencing. J. Basic. Microbiol..

[171] Ding Y., Yi Z., Fang Y., He K., Huang Y., Zhu H., Du A., Tan L., Zhao H., Jin Y. (2023). Improving the quality of barren rocky soil by culturing sweetpotato, with special reference to plant-microbes-soil interactions. Microbiol. Res..

[172] Jansson J.K., Hofmockel K.S. (2020). Soil microbiomes and climate change. Nat. Rev. Microbiol..

[173] Singh A.K., Singh P.K., Arya M., Singh N.K., Singh U.S. (2015). Molecular Screening of Blast Resistance Genes in Rice using SSR Markers. Plant Pathol. J..

[174] Zhou J., Luan X., Liu Y., Wang L., Wang J., Yang S., Liu S., Zhang J., Liu H., Yao D. (2023). Strategies and Methods for Improving the Efficiency of CRISPR/Cas9 Gene Editing in Plant Molecular Breeding. Plants.

[175] Li B., Sun C., Li J., Gao C. (2024). Targeted genome-modification tools and their advanced applications in crop breeding. Nat. Rev. Genet..

[176] Du M., Sun C., Deng L., Zhou M., Li J., Du Y., Ye Z., Huang S., Li T., Yu J. (2025). Molecular breeding of tomato: Advances and challenges. J. Integr. Plant Biol..

[177] Kumlehn J., Pietralla J., Hensel G., Pacher M., Puchta H. (2018). The CRISPR/Cas revolution continues: From efficient gene editing for crop breeding to plant synthetic biology. J. Integr. Plant Biol..

[178] Farooq M.A., Gao S., Hassan M.A., Huang Z., Rasheed A., Hearne S., Prasanna B., Li X., Li H. (2024). Artificial intelligence in plant breeding. Trends Genet..

[179] Qiu J., Tang G., Feng T., Zheng B., Liu Y., Zheng P. (2026). Transforming a fragile protein helix into an ultrastable scaffold via a hierarchical AI and chemistry framework. Elife.

[180] Ma Z., Gao W., Liu L., Liu M., Zhao N., Han M., Wang Z., Jiao W., Gao Z., Hu Y. (2020). Identification of QTL for resistance to root rot in sweetpotato (*Ipomoea batatas* (L.) Lam) with SSR linkage maps. BMC Genom..

[181] Rausher M.D. (2001). Co-evolution and plant resistance to natural enemies. Nature.

[182] Yang C., Liu R., Pang J., Ren B., Zhou H., Wang G., Wang E., Liu J. (2021). Poaceae-specific cell wall-derived oligosaccharides activate plant immunity via OsCERK1 during *Magnaporthe oryzae* infection in rice. Nat. Commun..

[183] Zhang X., Tang C., Jiang B., Zhang R., Li M., Wu Y., Yao Z., Huang L., Luo Z., Zou H. (2025). Refining polyploid breeding in sweet potato through allele dosage enhancement. Nat. Plants.

[184] Cao X., Xie H., Song M., Lu J., Ma P., Huang B., Wang M., Tian Y., Chen F., Peng J. (2022). Cut-dip-budding delivery system enables genetic modifications in plants without tissue culture. Innovation.

[185] Mei G., Chen A., Wang Y., Li S., Wu M., Hu Y., Liu X., Hou X. (2024). A simple and efficient *in planta* transformation method based on the active regeneration capacity of plants. Plant Commun..

[186] Wang H., Wu Y., Zhang Y., Yang J., Fan W., Zhang H., Zhao S., Yuan L., Zhang P. (2019). CRISPR/Cas9-Based Mutagenesis of Starch Biosynthetic Genes in Sweet Potato (*Ipomoea batatas*) for the Improvement of Starch Quality. Int. J. Mol. Sci..

[187] Tussipkan D., Manabayeva S.A. (2021). Employing CRISPR/Cas Technology for the Improvement of Potato and Other Tuber Crops. Front. Plant Sci..

[188] Tang W., Ma M., Song W., Kou M., Wang X., Yan H., Li C., Zhang A., Gao T., Gao R. (2026). An efficient CRISPR/Cas9-mediated editing of phytoene desaturase in hexaploid sweetpotato. Plant Sci..

[189] Peng K., Bai Y., Dai Z., Shang M., Zhang J., Zhai H., Gao S., Zhao N., Liu Q., He S. (2026). Snowwhite: A rapid genetic tool for sweet potato functional genomics. Plant Physiol..

[190] Ye X., Al-Babili S., Klöti A., Zhang J., Lucca P., Beyer P., Potrykus I. (2000). Engineering the Provitamin A (β-Carotene) Biosynthetic Pathway into (Carotenoid-Free) Rice Endosperm. Science.

[191] Zhu Q., Zeng D., Yu S., Cui C., Li J., Li H., Chen J., Zhang R., Zhao X., Chen L. (2018). From Golden Rice to aSTARice: Bioengineering Astaxanthin Biosynthesis in Rice Endosperm. Mol. Plant.

[192] Zhu X., Liu X., Liu T., Wang Y., Ahmed N., Li Z., Jiang H. (2021). Synthetic biology of plant natural products: From pathway elucidation to engineered biosynthesis in plant cells. Plant Commun..

[193] Su W., Xue H. (2021). Imaging Spectroscopy and Machine Learning for Intelligent Determination of Potato and Sweet Potato Quality. Foods.

[194] Al-Shamasneh A.R. (2025). Potato leaves disease classification based on generalized Jones polynomials image features. MethodsX.

[195] Jumper J., Evans R., Pritzel A., Green T., Figurnov M., Ronneberger O., Tunyasuvunakool K., Bates R., Žídek A., Potapenko A. (2021). Highly accurate protein structure prediction with AlphaFold. Nature.

[196] Abramson J., Adler J., Dunger J., Evans R., Green T., Pritzel A., Ronneberger O., Willmore L., Ballard A.J., Bambrick J. (2024). Accurate structure prediction of biomolecular interactions with AlphaFold 3. Nature.

[197] Li G., An L., Yang W., Yang L., Wei T., Shi J., Wang J., Doonan J.H., Xie K., Fernie A.R. (2025). Integrated biotechnological and AI innovations for crop improvement. Nature.

[198] Xie Y., Zhang T., Yang M., Lyu H., Zou Y., Sun Y., Xiao J., Lian W., Tao J., Han H. (2025). Engineering crop flower morphology facilitates robotization of cross-pollination and speed breeding. Cell.

[199] Ali M., Wang Z., Guo Q., Wang Y., Cai Y., Du J., Pi E., Ding P., Shen J. (2026). Mapping plant cell-type-specific responses to environmental stresses. Trends Plant Sci..

[200] Nybom H., Lacis G. (2021). Recent Large-Scale Genotyping and Phenotyping of Plant Genetic Resources of Vegetatively Propagated Crops. Plants.

[201] Zhong Y., Ahmed S., Deng G., Fan W., Zhang P., Wang H. (2019). Improved insect resistance against *Spodoptera litura* in transgenic sweetpotato by overexpressing *Cry1Aa* toxin. Plant Cell Rep..

[202] Chen S.P., Kuo C.H., Lu H.H., Lo H.S., Yeh K.W. (2016). The Sweet Potato NAC-Domain Transcription Factor IbNAC1 Is Dynamically Coordinated by the Activator IbbHLH3 and the Repressor IbbHLH4 to Reprogram the Defense Mechanism against Wounding. PLoS Genet..

